# Discovery and Preliminary Characterization of Lactose-Transforming Enzymes in *Ewingella americana* L47: A Genomic, Biochemical, and In Silico Approach

**DOI:** 10.3390/ijms27021128

**Published:** 2026-01-22

**Authors:** Katherine Rivero, Rodrigo Valenzuela, Inaira Rivero, Pedro General, Nicole Neira, Fernanda Contreras, Jans Alzate-Morales, Claudia Muñoz-Villagrán, Carlos Vera, Mauricio Arenas-Salinas, Felipe Arenas

**Affiliations:** 1 Laboratorio de Microbiología Molecular, Facultad de Química y Biología, Universidad de Santiago de Chile, Santiago 9170022, Chile; katherine.rivero@usach.cl (K.R.); inaira.rivero@usach.cl (I.R.); pedro.general@usach.cl (P.G.); nicole.neira@usach.cl (N.N.); claudia.munoz.v@usach.cl (C.M.-V.); carlos.vera.v@usach.cl (C.V.); 2 Centro de Bioinformática, Simulación y Modelado (CBSM), Facultad de Ingeniería, Universidad de Talca, Talca 3465548, Chile; rvalenzuelaperez1789@gmail.com (R.V.); jalzate@utalca.cl (J.A.-M.); marenas@utalca.cl (M.A.-S.); 3 Laboratorio de Ecología de Ambientes Extremos, Centro de Genómica, Ecología y Medio Ambiente (GEMA), Universidad Mayor, Santiago 8580745, Chile; fernanda.contreras.t@usach.cl

**Keywords:** lactose valorization, β-galactosidase, L-arabinose isomerase, D-tagatose, inclusion bodies, biocatalysis

## Abstract

D-tagatose is a high-value, low-calorie sweetener that can be produced from dairy lactose via a two-step enzymatic route: lactose hydrolysis to galactose followed by galactose isomerization to tagatose. Here, we combined genomics, in silico structural analysis, and biochemical assays to evaluate the lactose-to-tagatose conversion potential of an Antarctic isolate, L47, identified as *Ewingella americana* (NCBI accession SAMN54554459). Genome mining revealed one L-arabinose isomerase gene (*araA*) and three β-galactosidase genes (*bgaA*, *bglY*, *lacZ*), an uncommon combination in a single bacterium. Recombinant AraA was produced in *Escherichia coli* and biochemically characterized, showing Mn^2+^ dependence and measurable D-galactose isomerization, reaching ~18% tagatose from 100 mM galactose after 48 h under the tested conditions. In contrast, the β-galactosidases were predominantly recovered as insoluble aggregates in *E. coli*; therefore, β-galactosidase activity was assessed using washed inclusion-body preparations. Under these conditions, BgaA displayed the most consistent o-NPG hydrolyzing activity, whereas BglY and LacZ did not yield reproducible activity. Overall, our results identify BgaA as the most tractable lactose-hydrolyzing candidate from L47 in the current workflow and indicate that AraA performance is the principal bottleneck toward an efficient lactose-to-tagatose process, motivating future optimization at the enzyme and process levels.

## 1. Introduction

Processed foods constitute a significant portion of human diets worldwide, accounting for an estimated 50–90% of caloric intake in some populations [1]. The prevalence of industrially processed food has exacerbated environmental challenges by generating large quantities of agro-industrial waste [2]. One notable example is cheese whey, a lactose-rich byproduct of the dairy industry. In many regions, whey is disposed of into the environment, where its high organic content causes pollution of water bodies. Although some whey is repurposed (e.g., for protein concentrates and animal feed), in developing countries such as Chile it is still often discarded, posing a serious environmental burden [3]. Reducing such waste aligns with circular economy and bioeconomy principles, which aim to transform biological waste streams into high-value products [4,5]. Implementing a circular bioeconomy, however, requires efficient biotechnological solutions to convert low-value substrates like lactose into value-added compounds.

Lactose valorization—converting lactose into higher-value products—has drawn considerable interest as a strategy to mitigate whey waste. A particularly attractive target product is D-tagatose, a keto-hexose sugar that is nearly as sweet as sucrose but with ~40% fewer calories and a very low glycemic index. D-Tagatose also exhibits prebiotic properties, making it a promising functional sweetener for foods and pharmaceuticals [6]. An enzymatic pathway can be employed to convert lactose into tagatose. In the first step, β-galactosidase (β-gal, EC 3.2.1.23) hydrolyzes lactose into D-glucose and D-galactose. This step is already used industrially to produce lactose-free dairy products and galactooligosaccharides (GOS) from whey lactose. In the second step, L-arabinose isomerase (L-AI, EC 5.3.1.4) isomerizes the released D-galactose into D-tagatose. L-AIs are actively being researched for tagatose production processes [6]. Both enzymatic steps add value to the whey stream: β-galactosidases facilitate lactose removal and GOS synthesis, while L-AIs enables the generation of a low-calorie sweetener (tagatose) with potential health benefits.

Structurally, the *Escherichia coli* β-galactosidase (LacZ) is a well-characterized model enzyme. It is a homotetramer of ~465 kDa, with each ~116 kDa monomer contributing to the active site at subunit interfaces. The active site pocket contains key residues that coordinate lactose and catalyze its hydrolysis via a classic double-displacement (ping-pong) mechanism. Two glutamic acid residues act in tandem—one as a general acid to protonate the glycosidic oxygen, and another as a nucleophile to form a galactosyl-enzyme intermediate—facilitating breakdown of the lactose into monosaccharides [7]. These catalytic glutamates and surrounding residues are conserved among β-galactosidases from various species [8]. Lactose binding is further stabilized by multiple hydrogen bonds (in *E. coli* LacZ, interactions involve Asn99, His391, Glu461, Gln537, etc.) and hydrophobic stacking with active site tryptophans, as well as coordination to essential metal ions [7]. *E. coli* LacZ requires Mg^2+^ for maximal activity, likely because the metal ion helps stabilize the transition state and coordinate reactive water molecules; removal of Mg^2+^ significantly impairs catalysis [9]. In addition, a Na^+^ ion is known to bind near the lactose 6-hydroxyl in *E. coli* LacZ, enhancing activity, though sodium is not universally required in all β-galactosidases [7]. Many bacterial β-galactosidases are metalloenzymes to varying degrees—for example, some require divalent cations (Mg^2+^ or Mn^2+^) for optimal activity, and some possess structural metal sites that influence stability [10]. Industrial applications of β-galactosidases leverage these properties to achieve high activity under process conditions, for instance using thermostable or cold-active enzymes depending on the desired operation temperature.

L-Arabinose isomerase (L-AI), in contrast, typically functions as a homotetramer or hexamer of ~500–600 kDa total. L-AIs catalyzes the reversible isomerization of D-galactose to D-tagatose (as well as L-arabinose to L-ribulose in their native context) via an ene-diol mechanism. The active site of L-AI usually contains two catalytic acidic residues (Glu/Asp) and two histidines, arranged to facilitate proton transfer and aldose-ketose isomerization. Many L-AIs are metalloenzymes, requiring a divalent metal cofactor (often Mn^2+^) bound in the active site for activity—the metal ion helps polarize the substrate and stabilize the enediolate intermediate [6]. Optimal activity of L-AIs is often at slightly acidic to neutral pH (around 6–7.5) and moderate temperatures, though there is variability among species. D-Tagatose formation by L-AI is typically equilibrium-limited (equilibrium favors galactose ~3:1 over tagatose at 30–60 °C), so yields around 30–50% can be achieved at high substrate concentrations or with equilibrium-shift strategies (e.g., continuous product removal). Recent engineering efforts have also produced metal-independent L-AIs, which have mutations in the metal-binding sites allowing them to catalyze isomerization without added metal ions [11].

Cold-active enzymes offer significant advantages, as they not only catalyze reactions at low temperatures but also maintain high activity and reaction rates by lowering the activation energy required. They are characterized by their higher specific activity at low temperatures and a relatively low optimum catalysis temperature [12]. This facilitates the economical production of enzymes through high-yield, high-productivity, intensive fermentation processes [13].

In the search for these types of enzymes, psychrophilic or psychrotolerant microorganisms have been investigated. Some relevant enzymes derived from psychrophilic bacteria include one from *Alkalilactibacillus ikkense*, which maintains 60% of its activity at 0 °C, demonstrating transgalactosylation activity [14], and one from *Arthrobacter* sp., with a peak activity of 42% at 10 °C [15]. Similarly, *Rahnella inusitata* has shown between 41–62% hydrolysis activity at temperatures of 4–15 °C [16]. Among psychrotolerant bacteria, active L-AIs have been identified, such as one from *Arthrobacter* sp., with a maximum activity of 60% for isomerizing D-galactose at 30 °C, without the need for divalent ions [17], and one from *Shewanella* sp., which shows maximum activity between 15 and 35 °C, with a moderate need for divalent cations, achieving 16% D-galactose isomerization at 4 °C and 34% at 35 °C [18]. Finally, an L-AI from *Pseudoalteromonas haloplanktis* was found, which showed activity at 40 °C, although without specificity for the substrate D-galactose [19].

To advance lactose waste valorization, in this work we investigate two key enzymes from a novel bacterial source: β-galactosidase (for lactose hydrolysis) and L-arabinose isomerase (for tagatose production). Our focus was on an Antarctic isolate, strain L47, which was identified as a Gram-negative *Ewingella americana* from genomic analyses. Notably, the draft genome of L47 revealed one L-arabinose isomerase gene and three distinct β-galactosidase genes, an unusual multiplicity for a single isolate (most bacteria have only one or two β-gal genes). This raised the question of whether one of these β-galactosidases specializes in lactose degradation. We hypothesized that only one of the three β-gal enzymes is primarily responsible for lactose hydrolysis in L47. Therefore, the objectives of this study were: (i) to identify which of the three L47 β-galactosidases has the highest lactose-degrading activity, by combining bioinformatic predictions with heterologous expression and enzymatic assays, and (ii) to characterize the activity of the L47 L-arabinose isomerase for D-tagatose production. We employed a combination of genomic analysis, structural modeling, and in vitro experiments (enzyme expression, purification, and kinetic assays) to achieve these goals. Our integrated approach allowed us to correlate the enzymes’ structures with their function, and to pinpoint the most promising enzyme candidates for a lactose-to-tagatose conversion process.

## 2. Results

### 2.1. Identification of Lactose-Hydrolyzing Activity in L47 and Related Isolates

Of the 28 Antarctic bacterial isolates tested, 17 were able to grow in rich LB medium at 15 °C. In minimal M9 medium with glucose as the sole carbon source (also at 15 °C), all those 17 strains showed robust growth. However, only 13 of the 17 grew when L-arabinose was provided as the sole carbon source under the same conditions. To identify candidates possessing L-arabinose isomerase (L-AI), the 16S rRNA gene from each of these 13 strains was sequenced and compared against reference databases. This allowed preliminary taxonomic identification and an assessment of whether the *araA* gene (encoding L-AI) is present in related known genomes. Notably, only two isolates (designated L47 and R61) appeared to harbor an *araA* gene in their genome. The remaining isolates were identified as species of genera such as *Pseudomonas* and *Pelomonas*, which metabolize L-arabinose via alternative oxidative pathways (converting L-arabinose to L-2-keto-3-deoxyarabonate and then to α-ketoglutarate) rather than through L-AI. This explains their ability to grow on L-arabinose despite lacking a classical L-AI enzyme [20,21,22,23]. In contrast, the presence of a putative *araA* in L47 and R61 suggested that these two isolates employ the isomerase pathway, making them promising candidates for enzymatic lactose conversion.

Since isolates L47 and R61 possess a conventional *araA* gene, these two strains were selected to evaluate the capacity of their cell extracts as biocatalysts for L-arabinose isomerization (L-arabinose to L-ribulose) and for D-galactose isomerization (D-galactose to D-tagatose). To induce expression of the L-AI enzyme, L47 and R61 were each cultured in M9 minimal medium with L-arabinose as the sole carbon source. Growth curves for both cultures were generated (Supplementary Figure S1) to determine the time needed to reach exponential phase. Figure S1A shows that both L47 and R61 grew steadily on L-arabinose, and this analysis allowed pinpointing the mid-exponential growth time for each strain, when L-AI expression would presumably be highest. Cells were harvested at those time points and lysed to prepare crude extracts for enzymatic assays.

**Figure 1:**
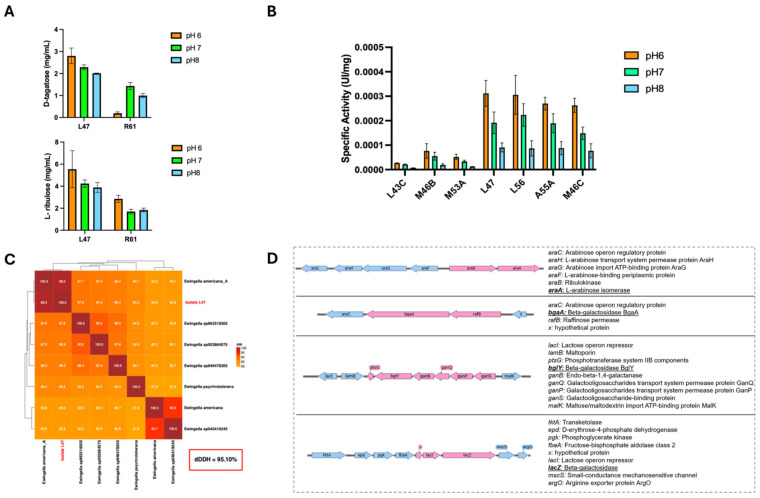
Determination of the enzymatic activity of β-Galactosidase and L-arabinose isomerase in crude extracts of environmental isolates and identification of these genes in isolate L47. (**A**) β-Galactosidase activity in crude extracts of environmental isolates to different pH using D-tagatose (up) and D-ribulose (down) as substrate by HPLC. (**B**) L-arabinose activity measured by o-NPG hydrolysis for environmental strains at different pH. (**C**) Average Nucleotide identity (ANI) in the 8 genome *Ewingella* dataset. The L47 strain forms a cluster with *Ewingella americana* with an alignment over 99%. (**D**) Genetic context of the loci containing the *araA*, *bgaA*, *bglY*, and *lacZ* genes, identified by bioinformatic analysis as operon-like modules. Genes shown in pink correspond to the CDSs proposed to be part of the operon, whereas genes shown in light blue correspond to the surrounding genomic context. Arrows indicate the transcriptional orientation of the genes.

After obtaining and quantifying the crude extracts from L47 and R61, the production of L-ribulose (from L-arabinose) and D-tagatose (from D-galactose) was measured under varying pH and temperature conditions. Figure 1A presents the results of the pH profile, while Figure S1B,C show the temperature profile for these isomerization reactions. Figure 1A (upper panel) shows the yield of L-ribulose produced by each extract across pH 6 to 8, and Figure 1A (lower panel) shows the corresponding yield of D-tagatose under the same pH range. Both L47 and R61 extracts were active in catalyzing the isomerization reactions between pH 6 and 8, but the optimal pH differed slightly between the strains. The L47 extract achieved its highest production of both L-ribulose and D-tagatose at pH 6, whereas the R61 extract showed its maximum L-ribulose production at pH 6 and its highest D-tagatose production at pH 7. In other words, L-AI enzyme of L47 prefers a slightly more acidic condition for both substrates, while enzyme R61 performs best at a neutral pH for the galactose-to-tagatose conversion. Figure S1B,C illustrate the effect of reaction temperature on L-ribulose and D-tagatose formation, respectively, by the two extracts. Both enzymes of the isolates were active over a broad temperature range. The data indicates that extract of L47 generally produced higher amounts of product at elevated temperatures compared to extract of R61. For example, L47 maintained substantial isomerization activity up to the higher temperatures tested, whereas R61 showed a more pronounced drop-in activity at those temperatures (as evidenced in Figure S1B,C). These results suggest that the L-AI from L47 may have a higher thermal optimum or stability than that of R61, an important consideration for potential industrial applications. In summary, the crude extracts of both L47 and R61 can convert L-arabinose to L-ribulose and D-galactose to D-tagatose, but L47 showed a stronger performance at pH 6 and maintained activity better at higher temperatures.

In parallel with the arabinose utilization tests, we examined the ability of the isolate to metabolize lactose. Eighteen out of the 28 isolates were found to grow in M9 minimal medium with lactose as the sole carbon and energy source (at 15–20 °C). This indicates that most of the Antarctic isolates retained some capacity for lactose utilization. From these lactose-positive strains, seven isolates displaying the fastest growth rates on lactose were chosen for more detailed analysis. These seven were strains M46C, M53A, A55A, M46B, L43C, L56, and L47. Notably, L47 was among the top performers in terms of growth on lactose, further highlighting its biotechnological potential. To verify that these strains indeed express β-galactosidase (the enzyme responsible for lactose hydrolysis), a chromogenic X-Gal plate assay was conducted. Each selected strain was streaked onto solid M9 minimal medium containing lactose (2% *w*/*v*) and the indicator compound X-Gal (5-bromo-4-chloro-3-indolyl-β-D-galactopyranoside). After incubation, six of the seven strains formed blue-colored colonies on the X-Gal plates (Figure S1D), confirming β-galactosidase activity in those isolates. The blue color arises from the cleavage of X-Gal by β-galactosidase, which releases an insoluble blue dye in the colony. In contrast, one of the seven strains did not produce a blue color on X-Gal, suggesting that it either lacks β-galactosidase or does not express it under these conditions. The X-Gal test thus qualitatively demonstrated that all but one of the fast-growing lactose-utilizing isolates possess active β-galactosidase, consistent with their ability to hydrolyze lactose into glucose and galactose for growth.

To quantitatively compare lactose metabolism among the selected strains, growth curves were measured in liquid M9 minimal medium with 2% *w*/*v* lactose at 20 °C for each of the seven isolates. Figure S1E shows the growth profiles (OD_600_ vs. time) for these cultures, highlighting differences in growth rates and lag times. Strain A55A exhibited the most rapid growth, reaching stationary phase in roughly 24 h, whereas L47 had a longer lag and attained stationary phase at approximately 30–35 h. The other strains showed intermediate growth dynamics (for instance, L56 and M46C grew faster than L47 but not as fast as A55A). Based on these growth curves, we determined appropriate harvest times for each strain such that cells could be collected in mid-exponential phase for enzyme extraction. In practice, each culture was stopped at the time point just before the onset of stationary phase (as indicated by Figure S1E) to ensure that β-galactosidase expression was active when the cells were harvested. This strategy maximized the enzyme yield and activity in the subsequent crude extracts.

Crude cell extracts were prepared from each of the seven selected strains, and their β-galactosidase activity was assayed under different pH conditions. Specifically, enzyme activities were measured at pH 6.0 to 8.0 using a standard β-galactosidase substrate (o-nitrophenyl-β-D-galactopyranoside, o-NPG) in the presence of 0.1 mM Mn^2+^ and 0.1 mM Ca^2+^ (these divalent cations were included because some β-galactosidases, especially cold-adapted or unusual ones, may require metal ions for optimal activity). Figure 1B summarizes the β-galactosidase activity of each strain’s extract at the three pH values (with both Mn^2+^ and Ca^2+^ present). The results demonstrate that four strains—L47, L56, A55A, and M46C—have markedly higher β-galactosidase activities compared to the other three strains (L43C, M53A, and M46B). At the optimal pH of 6, these top four strains showed significantly greater o-NPG hydrolysis rates (indicative of lactose-hydrolysis capability) than the lower-performing isolates. All seven enzymes exhibited a clear pH optimum around 6. Enzyme activity dropped as the pH was raised to neutral (7) and alkaline (8) conditions, and at pH 8 most of the strains retained only a small fraction of their activity. Thus, pH 6 was found to be the optimal condition for β-galactosidase activity in all the environmental isolates tested (Figure 1B), which is consistent with many β-galactosidases preferring slightly acidic conditions.

Finally, the temperature dependence of β-galactosidase activity was examined for the three most active isolates (L47, A55A, and M46C). Crude extracts of these strains were assayed over a broad temperature range from 4 °C up to 60 °C, to assess both low-temperature activity and thermal stability. Figure S1F presents the enzyme activity profiles (relative activity vs. temperature) for L47, A55A, and M46C. The L47 extract stood out for its high activity across a wide range of temperatures. Remarkably, β-galactosidase activity of L47 did not decrease with increasing temperature throughout the range; instead, it continued to climb, reaching its maximum activity at 60 °C, the highest temperature tested. Even at 50–60 °C, L47 maintained the highest specific activity among the three, indicating a high level of thermal tolerance or adaptation. In contrast, the enzymes from A55A and M46C were more cold-active: they exhibited much lower optimum temperatures, with the activity of A55A peaking around 15 °C and M46C around 20 °C. These two strains showed declining activity above their optimal temperatures, and by 60 °C their enzyme activities were minimal. Another notable difference was observed at the low end of the temperature spectrum (4 °C). At 4 °C, extract of L47 retained only ~16% of its maximum activity (relative to the 60 °C peak), whereas A55A and M46C retained approximately 53% and 71% of their maximal activities (which were achieved at 15–20 °C), respectively. In practical terms, this means that the β-galactosidase of L47 is more effective at higher temperatures but loses most of its activity in the cold, whereas the enzymes from A55A and M46C are adapted to work well at low temperatures (retaining most of their activity at 4 °C) but are unstable or inactive at 50–60 °C. Despite these differences, L47 demonstrated the highest absolute activity values at nearly every temperature tested, making it the superior enzyme candidate overall.

In summary, the comparative screening of environmental isolates revealed that strain L47 possesses exceptional biocatalytic capabilities for lactose conversion. Its crude extracts not only showed the ability to isomerize D-galactose into D-tagatose and L-arabinose into L-ribulose, but also consistently exhibited the highest β-galactosidase activities across a range of pH and temperature conditions. Given that L47 excelled in both key activities (lactose hydrolysis and galactose isomerization) critical for our lactose-to-tagatose conversion goal, we selected isolate L47 for further detailed study. Subsequent sections focus on the genomic characterization of L47 and the purification and characterization of its lactose-converting enzymes, capitalizing on the superior performance demonstrated by this strain in the initial screenings.

### 2.2. Genome Sequencing and Bacterial Identification of L47 Isolate

Given the superior enzyme activities exhibited by strain L47 (see Section 2.1), this Antarctic isolate was selected for whole-genome sequencing to determine its precise taxonomic identity and genetic capacity. High-quality genomic DNA of L47 was extracted and sequenced (Plasmidsaurius), and the draft genome was deposited in NCBI under accession number SAMN54554459. In silico comparisons confirmed that L47 belongs to *Ewingella americana*, a psychrotolerant Gram-negative species. In particular, the L47 genome shares over 99% average nucleotide identity (ANI) with reference *E. americana* genomes (Figure 1C) and a digital DNA–DNA hybridization (dDDH) value of 95.10%, well above species-demarcation thresholds. These metrics firmly establish L47 as *E. americana*. Genomic analysis further revealed that L47 harbors four key catabolic genes not typically found together in one organism: one L-arabinose isomerase gene and three distinct β-galactosidase genes. L-arabinose isomerase gene (*araA*) is 1506 bp (501 aa, ~56 kDa) while the β-galactosidase genes (designated *bgaA*, *bglY*, and *lacZ*) are approximately 2007 bp (668 aa, ~75 kDa), 2061 bp (686 aa, ~77 kDa), and 3201 bp (1033 aa, ~110 kDa) in length, respectively. BLAST analysis confirmed that each of these genes is nearly identical (99–100% nucleotide identity) to sequences from *E. americana* in public databases. Notably, the putative LacZ protein shares high homology with the canonical GH2-family β-galactosidase of *E. coli*, whereas BgaA and BglY belong to the GH42 family of β-galactosidases commonly found in *Bacillus* and other Gram-positive bacteria. Sequence alignment also indicated that BgaA and BglY are only ~58% identical to each other, confirming they are distinct isozymes rather than recent gene duplicates. Intriguingly, these genes are not organized in L47 as classical sugar operons. The identification of three genes encoding β-galactosidases in the genome of strain L47 represents an unusual feature, as most bacteria harbor only one or two genes of this type. This observation suggests that these enzymes may fulfill distinct physiological roles or be expressed under different environmental conditions, which motivated a detailed analysis of loci associated with carbohydrate metabolism. Automatic annotation of the L47 genome using Prokka estimated an approximate genome size of ~4.88 Mb, comprising 4435 protein-coding genes (CDS), and identified the *rpoD* gene, which encodes the housekeeping sigma factor σ^70^, together with several alternative sigma factors. Integration of structural annotation (Prokka) and transcriptional unit prediction using Operon-mapper enabled the identification of four carbohydrate metabolism-associated loci containing the *araA*, *bgaA*, *bglY*, and *lacZ* genes (Figure 1D). These loci exhibited CDS collinearity, conserved transcriptional orientation, and short intergenic distances, with predicted operon probabilities ranging from 0.86 to 0.98. Analysis of upstream regions using BPROM identified at least one putative promoter per locus, featuring −35/−10 boxes compatible with σ^70^-dependent promoters and Linear Discriminant Function (LDF) values between 2.41 and 4.65. Overall, examination of the genomic context of the *araA*, *bgaA*, *bglY*, and *lacZ* loci revealed the association of catabolic genes with carbohydrate transport systems and specific transcriptional regulators (AraC- and LacI-like), an organization characteristic of inducible operons. This architecture suggests that these loci may be transcribed by σ^70^-associated RNA polymerase, with regulation modulated by carbon source availability. This finding raised the possibility that one β-galactosidase could be specialized for lactose hydrolysis in L47, a hypothesis investigated in later experiments.

In addition to genomic characterization, the identity of L47 was supported by morphological (Figure S2) and biochemical analyses (Supplementary Table S1). Microscopic examination confirmed that L47 is a Gram-negative rod. In particular, a Gram stain showed rod-shaped cells (Figure S2A), and scanning electron microscopy revealed bacilli approximately 1.8–2.0 μm in length (Figure S2B). Biochemical profiling was carried out using the RapID™ ONE panel for Gram-negative bacteria. The metabolic fingerprint of L47 (Table S1) was analyzed with RapID ERIC software (version v.1.0.771), which mis-identified the isolate as *Serratia plymuthica* with a calculated probability > 99.9%. This result contrasts with the genome-based identification of L47 as *Ewingella americana*. The discrepancy is attributable to the close phylogenetic and phenotypic relatedness among *Ewingella* and certain *Serratia*-lineage bacteria. Indeed, *E. americana* is known to cluster within the *Serratia*/*Yersinia* branch of the Enterobacteriaceae in 16S rRNA phylogenies. Prior studies have noted that *Ewingella* can exhibit biochemical traits overlapping with those of *Serratia*, complicating identification by kit-based tests. Spröer et al. (1999) [24] demonstrated that *Ewingella americana* falls into a clade alongside *Serratia*, *Yersinia*, and *Rahnella* species. Thus, while genomic ANI/dDDH analysis unambiguously places L47 in the *Ewingella* clade, its conventional biochemical profile can appear deceptively *Serratia*-like. In summary, the genomic and phenotypic evidence together establish L47 as a member of *Ewingella americana*, carrying an uncommon repertoire of three β-galactosidases and one L-arabinose isomerase that are dispersed in the genome rather than arranged in classic operons. This unique genetic makeup prompted further investigation into the function of each enzyme in the context of lactose metabolism and D-tagatose production by L47.

### 2.3. In Silico Structural Analysis (Homology Models, Docking, and Dynamics)

**Figure 2:**
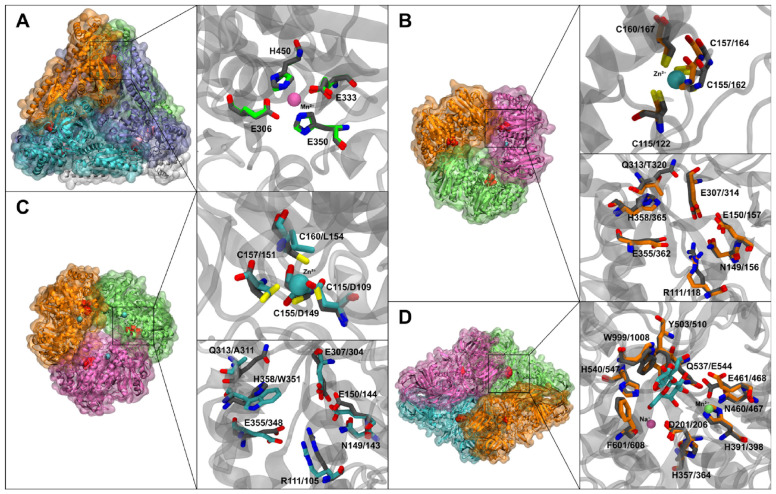
Homology models of L47 enzymes and key active-site features. In all panels, residues shown in gray correspond to the template structure used for homology modeling. Residues corresponding to AraA, BglY/LacZ, and BgaA are shown in green, orange, and cyan, respectively (**A**) AraA model. The catalytic metal (Mn^2+^, purple sphere) is coordinated by conserved residues. The catalytic glutamates (Glu333, Glu306 in this model) and histidines are positioned as in the *E. coli* AraA structure. (**B**) BgaA model superimposed on a *Bacillus* GH42 template. The active site is highlighted: note the Trp351 (orange stick) replacing a conserved His, and Ala311 replacing Gln. The Zn-binding loop (inset) has Cys→Asp/Leu substitutions and is unable to coordinate Zn^2+^ (modeled Zn shown as translucent cyan sphere with missing Cys bonds). (**C**) BglY model. All four cysteines of the zinc-finger (orange sticks for L47, gray sticks for template model) are intact, coordinating a Zn^2+^ (cyan sphere). The active-site residues overlay almost exactly with the template (only Thr320 is ifferent, circled). (**D**) LacZ model shows the large β-galactosidase monomer. The active site contains a Mg^2+^ (green sphere), Na^+^ (magenta sphere) and the docked lactose in cyan. The two catalytic glutamates (Glu461, Glu645) and other binding residues are conserved. The unique Glu544 substitution in L47 LacZ is near the active site, introducing an extra negative charge. Although the overall folds are similar, local differences at the active site and metal-binding regions distinguish these enzymes functionally. Ligands: lactose shown in cyan in Figure 2D; for clarity, not all protein side chains shown.

Prior to measuring enzyme kinetics, we analyzed the enzyme structures in silico to predict which β-galactosidase might be most efficient. Figure 2 presents the homology models of AraA, BgaA, BglY, and LacZ, highlighting key active-site features.

Figure 2A shows the model of L47 AraA (teal) superimposed on the hexameric *E. coli* AraA template. The model suggests AraA forms a hexamer (though for clarity only one subunit is shown with the active site). A Mn^2+^ (purple sphere) is coordinated in the active site by conserved residues (Glu, Asp, His, as in template structure PDB ID: 2AJT). The catalytic glutamates (modeled as Glu332 and Glu346 in L47 AraA) and histidines (His104, His173) align well with those in *E. coli* AraA, indicating a typical active site for aldose-ketose isomerization [25].

Figure 2B shows the model of L47 BgaA (green), which was built on a GH42 template structure PDB ID: 3TTS (a *Bacillus circulans* β-galactosidase). BgaA is predicted to form a homotrimer (like other GH42 enzymes). The active site of BgaA is highlighted in gray for L47 and orange for the modeller model. Notably, BgaA has a tryptophan (Trp351, orange stick) where the template enzyme has a conserved histidine, and an alanine (Ala311) where a conserved glutamine would be [26]. These substitutions are near the sugar-binding pocket (inset of Figure 2B). The loss of the histidine at position 351 means one less potential hydrogen bond to lactose (the His in other GH42 enzymes can interact with the substrate’s O4/O3). Trp351, being bulkier and hydrophobic, might alter the binding geometry, possibly reducing affinity slightly but perhaps improving hydrophobic packing. The Zn-binding loop in BgaA (which in other GH42 enzymes coordinates a structural Zn^2+^) is mutated: as mentioned, three of four cysteines are replaced (indicated in red). In our model, we could not dock a Zn^2+^ in BgaA because the cysteine ligands are absent (the cyan sphere indicates where Zn would be if the loop were intact). This suggests that structure of BgaA might be somewhat more flexible or less stable in that region, as it cannot be reinforced by a zinc ion.

Figure 2C displays L47 BglY model of BglY, based on the same GH42 template structure PDB ID: 3TTS, and it shows a fully intact zinc-finger subdomain (blue sticks indicate the four cysteine residues). We included a Zn^2+^ (blue sphere) in the model, which is tetrahedrally coordinated by those cysteines, likely stabilizing a loop adjacent to the active site [26]. The active-site residues of BglY overlay almost exactly with the template’s catalytic residues. Only a single substitution, Gln→Thr at position 320 (circled in Figure 2C), is observed near the active site; this change is relatively minor (threonine is smaller but still polar) and is not expected to drastically affect lactose binding. Thus, structurally, BglY appears very similar to other active GH42 β-galactosidases, and one would predict it to be a functional lactose hydrolase. The presence of the Zn^2+^-binding site suggests that if Zn^2+^ is available, BglY could have enhanced stability or activity [27].

Figure 2D shows L47 LacZ. The model of LacZ (monomer) aligns closely with *E. coli* LacZ obtained from template structure 1JYN (RMSD ~0.6 Å over core domains), confirming that L47’s LacZ is a typical GH2 β-galactosidase. The active site is very well conserved, containing a bound Mg^2+^ (green sphere) modeled after the known position in *E. coli* LacZ, and a nearby Na^+^ site (not explicitly modeled, but would be near a galactose 6-hydroxyl). The hydrogen bonds related to magnesium are mostly mediated by water molecules, which interact with lactose and with amino acids such as Asp206 and Glu468, numbering based on strain L47. The two catalytic glutamates (Glu461 and Glu645 in L47 numbering, equivalent to Glu537 and Glu461 of *E. coli* LacZ) are present and positioned correctly. One unique difference is Glu544 in L47 LacZ (colored in gray sticks Figure 2D for template model and orange sticks for L47), which replaces a glutamine (Gln537 in *E. coli* LacZ). Aside from that substitution, all key binding residues (Asn102, His418, Phe601, etc., numbering based on strain L47) are present. Therefore, if L47 LacZ can be expressed and folded properly, we would expect it to be a highly active β-galactosidase, much like *E. coli* LacZ.

In summary, the structural models pointed to a few hypotheses: (i) LacZ should have very strong lactose-binding (as in *E. coli* LacZ) and thus high potential activity, provided it can be produced; (ii) BglY has a complete active site and structural Zn^2+^, indicating it should also be an active β-gal, possibly benefiting from Zn-mediated stability; (iii) BgaA lacks the Zn^2+^ site and has two active-site mutations, which might reduce its lactose affinity or stability, potentially making it less active than BglY; and (iv) AraA is a typical metal-dependent isomerase, likely functional given its conserved catalytic residues.

### 2.4. Molecular Dynamics and Binding Affinity Predictions

To complement the structural models, we performed molecular dynamics (MD) simulations for each enzyme–substrate complex: LacZ–lactose, BgaA–lactose, BglY–lactose, and AraA–galactose. All systems remained stable over 100 ns, with backbone RMSDs stabilizing between 2.5 and 3.5 Å (Figure S3). Lactose remained bound in the active sites of LacZ, BgaA, and BglY throughout the simulations. In LacZ and BglY, the distance between the catalytic glutamate Oε and the lactose glycosidic oxygen remained close to 3 Å, suggesting stable substrate positioning. In BgaA, occasional fluctuations increased this distance to ~4 Å due to movement of the loop containing Trp351, suggesting slightly looser—but still retained—binding. In the AraA–galactose complex, galactose remained near the catalytic center, coordinated to Mn^2+^ via water molecules, with the system showing overall RMSD stability around 2.8 Å.

The stability of key catalytic residues was further evaluated using RMSF analysis. Residues Glu160, Glu35, Trp351, and Thr320 displayed low fluctuations, indicating structural stability of the active site throughout the simulation (Figure S4).

To qualitatively compare substrate binding across the L47 enzymes, we estimated binding free energies using MM/GBSA calculations from equilibrated MD trajectories (Table 1). These analyses, performed on homology models, are meant as qualitative indicators rather than absolute measures of binding affinity. The results suggested relatively strong predicted binding of lactose to LacZ (ΔG_bind_ ≈ −28.3 kcal/mol) and BgaA (ΔG_bind_ ≈ −24.7 kcal/mol), weaker interaction for BglY (ΔG_bind_ ≈ −7.4 kcal/mol), and moderate affinity of AraA for D-galactose (ΔG_bind_ ≈ −12.5 kcal/mol).

It is important to note that these values do not consider factors such as folding efficiency, oligomeric state, or metal coordination, which may critically influence activity. For example, BglY functions as a trimer, and its full active-site architecture likely depends on structural elements not captured in the monomeric MM/GBSA setup.

These computational analyses were used exclusively for hypothesis generation and to prioritize experimental efforts. As demonstrated in our biochemical assays, predicted binding trends did not directly correlate with enzymatic activity. This highlights the limitations of in silico predictions and underscores the necessity of experimental validation when evaluating enzyme function.

### 2.5. Overexpression and Preparation of L47 Enzymes

**Figure 3:**
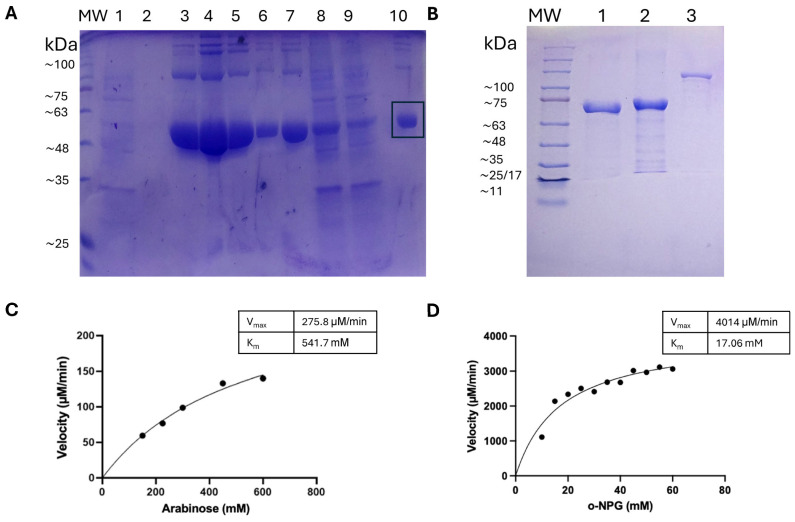
SDS-PAGE and enzymatic performance of purified L47 enzymes. (**A**) Stages of purification of the *E. americana* L-arabinose isomerase protein expressed in *E. coli* Rosetta (DE3) using 0.5 mM IPTG. PM: molecular weight marker (kDa); 1–2: first and second binding buffer washes; 3–7: eluted fractions 1–5 from HiTrap column; 8: post-sonication cell lysate; 9: flow-through after protein loading; 10: purified L-AI protein. The black box indicates the band corresponding to the target protein. (**B**) Production of β-galactosidase proteins of *E. americana* as washed inclusion bodies in *E. coli* BL21 (DE3) induced with 1 mM IPTG. MW: Molecular weight marker (kDa); 1: BgaA; 2: BglY; 3: LacZ. (**C**) Michaelis–Menten curve for AraA with L-arabinose as substrate (pH 7.5, 30 °C). Initial velocities (µM L-ribulose/min) are plotted against L-arabinose concentration (50–600 mM). (**D**) Michaelis–Menten curve for BgaA with o-NPG as substrate (pH 6, TA, 1 mM MgCl_2_) using inclusion bodies. Initial velocities (µM o-NP/min) are plotted against o-NPG concentration (10–60 mM). Data points (mean of three replicates) are fit to the Michaelis–Menten model (solid line). The kinetic parameters obtained are indicated in the inset.

We successfully overexpressed the L47 enzymes in recombinant *E. coli* hosts. AraA (~56 kDa) was predominantly expressed in soluble form and was purified using standard protocols from *E. coli* Rosetta cells (Figure 3A). In contrast, the β-galactosidases BgaA, BglY, and LacZ (approximately 75, 72, and 110 kDa, respectively) accumulated as inclusion bodies (Figure 3B). Several solubilization and refolding protocols were tested for these enzymes; however, they consistently failed to recover active protein or led to severe activity loss. Therefore, only washed inclusion body preparations were used for subsequent enzymatic activity assays. Washed inclusion bodies of BgaA and BglY were obtained in moderate yields (~5 mg per liter of culture), while LacZ was recovered in smaller amounts. SDS-PAGE analysis showed that both the purified AraA and the β-galactosidases from inclusion bodies appeared as single bands (Figure 3A,B), indicating good expression levels. However, only BgaA showed detectable enzymatic activity in the washed inclusion body preparations. LacZ and BglY showed no measurable activity, likely due to misfolding or aggregation in the inclusion state.

### 2.6. Activity of Recombinant Enzymes

β-Galactosidase activity from washed inclusion bodies: To determine which of the L47 β-galactosidase candidates was functionally active, we assessed o-NPG hydrolysis using washed inclusion body preparations from *E. coli* expressing each gene. Among them, only BgaA consistently exhibited measurable activity, while BglY and LacZ showed very low or background-level activity. Figure S5 summarizes the β-galactosidase activity profiles from these preparations across pH 4–7. At pH 6, BgaA showed the highest activity, approximately 0.0005 U per mg of total protein, about five times higher than BglY and LacZ (~0.0001 U/mg or less) (Figure S5). Activity decreased at pH 7 for all samples, but BgaA still retained the lead. These results suggest that only BgaA retained partial catalytic function in its aggregated form and that LacZ and BglY were largely inactive, possibly due to misfolding or expression-related limitations. All assays included Mn^2+^, and the relative activity trend remained consistent, suggesting that the higher activity of BgaA was not solely due to metal ion effects but reflected intrinsic properties of the protein. Although we cannot fully exclude low residual activity in LacZ or BglY, the data support BgaA as the primary lactose-hydrolyzing enzyme from L47 capable of partial activity in a recombinant context without proper refolding.

L-Arabinose isomerase activity and tagatose production: We next examined the performance of purified AraA in converting D-galactose to D-tagatose. Kinetic parameters for its native substrate, L-arabinose, were determined at pH 7.5 and 30 °C. The Michaelis–Menten curve (Figure 3C) yielded a Vmax of approximately 276 µM/min per mg enzyme and an apparent K_m_ of ~542 mM for L-arabinose. This very high K_m_ indicates low substrate affinity, which is consistent with other bacterial L-arabinose isomerases that typically exhibit K_m_ values in the 100–500 mM range for arabinose or galactose [6]. The catalytic efficiency is thus low, and high substrate concentrations would be required for significant activity. To evaluate tagatose production, AraA (0.1 mg/mL) was incubated with 100 mM D-galactose in the presence of Mn^2+^ at 30 °C for 48 h. HPLC analysis revealed a yield of ~1.8 mg/mL D-tagatose, corresponding to ~18% conversion. When the initial galactose concentration was reduced to 70 mM, the conversion dropped to ~3% (~0.38 mg/mL tagatose). These yields are relatively low compared to a recently reported metal-independent L-arabinose isomerase from *Bacillus amyloliquefaciens*, which achieved ~47% yield from 100 g/L (~0.55 M) galactose without requiring metal cofactors [11]. The modest performance of L47 AraA may be attributed to the lower substrate concentrations used in our assays and the high K_m_ of the enzyme. The theoretical equilibrium yield for galactose isomerization at 30 °C is approximately 30–40% [6], suggesting that our 18% yield either reflects an incomplete reaction or enzyme instability during the incubation. Indeed, partial precipitation of AraA was observed after 48 h at 30 °C, indicating some loss of activity over time.

We also tested AraA activity across pH values and metal cofactors. The enzyme was most active at pH ~6, with activity decreasing at pH 7 and becoming nearly undetectable at pH 8 (Figure S5). Regarding metal dependence, AraA was strictly dependent on divalent metal ions. No tagatose was produced in the absence of metal. Mn^2+^ supported the highest activity (normalized to 100%), followed by Co^2+^ (~50% activity), while Mg^2+^ was considerably less effective. Combining Mn^2+^ and Co^2+^ did not improve activity beyond Mn^2+^ alone. These results confirm that L47 AraA is a classical metal-dependent L-arabinose isomerase, similar to *E. coli* AraA and distinct from engineered metal-independent variants [11]. Its moderate thermostability and high substrate requirement suggest that, in its native form, the enzyme may not be optimal for industrial-scale tagatose production without further engineering or process optimization.

Kinetic evaluation of BgaA from inclusion bodies: Since BgaA showed the highest β-galactosidase activity among the tested enzymes, we performed a kinetic analysis using washed inclusion body preparations. Although the enzyme was not purified or properly refolded, it retained sufficient activity to allow for o-NPG kinetic assays at pH 6 and 37 °C (Figure 3D). The reaction followed Michaelis–Menten kinetics, yielding a V_max_ of ~4014 µM/min and an apparent K_m_ of ~17.1 mM for o-NPG. This corresponds to a specific activity of approximately 4.0 U/mg of total protein in the washed inclusion body sample. While these values are lower than those typically obtained from purified enzymes, they suggest that BgaA retains partial native conformation and catalytic activity, even in its aggregated state.

Similar assays were attempted for BglY and LacZ, but no reliable kinetic parameters could be obtained due to negligible activity. These findings reinforce BgaA as the only β-galactosidase from L47 exhibiting detectable catalytic function under the conditions tested, in line with computational predictions indicating lower binding affinities for BglY and the poor expression and folding observed for LacZ.

## 3. Discussion

In this study, we combined genomic, structural, and biochemical approaches to evaluate the lactose-to-tagatose conversion potential of the Antarctic isolate L47. Structural models are discussed exclusively in terms of sequence conservation and putative active-site architecture of the native enzymes, as the recombinant β-galactosidases were obtained predominantly as inclusion bodies. Therefore, any structural interpretation is limited to comparative and predictive insights rather than direct confirmation of native quaternary organization.

Genomic annotation of strain L47 revealed an operon-like organization for *araA*, *bgaA*, *bglY*, and *lacZ* loci, with putative −35/−10 motifs compatible with σ^70^-dependent promoters, suggesting basal transcription driven by the housekeeping RNA polymerase–RpoD (σ^70^). However, the presence and arrangement of nearby transcriptional regulators indicate that expression is primarily inducible and governed by carbon source availability, consistent with classical operon models [28]. The *ara* region exhibits a canonical L-arabinose-inducible architecture consistent with AraC-dependent activation [29], whereas the *rafB*–*bgaA* module couples transport and lactose hydrolysis and is flanked by an AraC-type regulator, supporting positive inducible control [30,31]. In contrast, the *bglY*–*gan* region integrates hydrolytic functions and transport with LacI-type regulators, consistent with a unit specialized in utilization of more complex galactosides, while the *lacI*–*lacZ* locus resembles a lac-type inducible system responsive to β-galactosides [32]. Collectively, these features support a regulatory network in which basal σ^70^ transcription is fine-tuned by dedicated regulators according to carbon source availability, consistent with the metabolic adaptability expected for environmental strains.

A central finding of this work is that, among the three β-galactosidases encoded in the L47 genome, BgaA displayed significant lactose-hydrolyzing activity, whereas the classical LacZ enzyme and the second GH42 β-galactosidase BglY showed little or no detectable activity under the experimental conditions tested. This result was initially unexpected, as in silico analyses predicted LacZ to exhibit the strongest lactose binding affinity, followed by BgaA, with BglY ranking lowest. Ultimately, the experimental results partially aligned with these predictions: BglY was indeed the least effective enzyme, while BgaA outperformed it. The main discrepancy arose from LacZ, which could not be obtained in an active form despite its favorable predicted binding properties, underscoring the importance of expression context and protein aggregation behavior in determining functional enzyme availability.

The differential activity observed between BgaA and BglY is particularly intriguing, given that both enzymes belong to the GH42 family and originate from the same organism. Several factors may explain this divergence. First, intrinsic differences in folding propensity and aggregation behavior likely play a major role. Although both enzymes accumulated as inclusion bodies in *E. coli*, BgaA-derived inclusion bodies consistently retained detectable catalytic activity, whereas BglY-derived aggregates did not. This observation suggests that BgaA inclusion bodies may contain a higher fraction of correctly folded or catalytically competent protein, a phenomenon increasingly reported for recombinant enzymes produced as inclusion bodies [33,34,35].

A second important distinction between BgaA and BglY relates to structural zinc dependence. Sequence and structural analyses indicate that BglY retains a canonical zinc-binding loop, likely involved in stabilizing the active-site architecture under native conditions [27]. In contrast, BgaA lacks key cysteine residues required for Zn^2+^ coordination, rendering it effectively zinc independent. Previous studies have demonstrated that Zn^2+^ supplementation can significantly enhance the activity of certain GH42 β-galactosidases when zinc is limiting [27]. In the present work, Zn^2+^ was not included in the activity assays, which may have disproportionately affected BglY functionality. Conversely, the apparent Zn^2+^ independence of BgaA likely confers greater robustness under heterologous expression conditions, allowing it to retain activity even within aggregated states.

Differences at the active-site level may further contribute to the observed behavior. Structural modeling suggested modest substitutions in BgaA relative to BglY, including His→Trp and Gln→Ala changes within the substrate-binding pocket. While these alterations could slightly affect substrate affinity, they do not appear to abolish catalytic function, as evidenced by the measured kinetic parameters for BgaA (K_m_ ≈ 17 mM for o-NPG). Such substitutions may even enhance hydrophobic packing or confer greater conformational flexibility, partially compensating for the loss of specific hydrogen bonds. In contrast, the inactivity of BglY is more plausibly attributed to structural instability and aggregation-related constraints rather than to active-site substitutions alone.

From a physiological perspective, it is also conceivable that BgaA and BglY fulfill distinct roles in the native L47 organism. Many bacteria encode multiple β-galactosidases with nonredundant functions, differing in substrate specificity, cellular localization, or induction conditions [31]. BgaA may represent the primary lactose-hydrolyzing enzyme expressed under lactose-rich conditions, whereas BglY could be induced under alternative environmental cues or act on different galactoside substrates. Without in vivo expression data, this remains speculative; however, our biochemical results strongly suggest that BgaA is the physiologically relevant lactose-active β-galactosidase in L47.

The case of LacZ further illustrates the gap between predicted enzymatic potential and practical applicability. Despite its favorable predicted lactose-binding affinity and the well-established efficiency of LacZ-type enzymes, the L47 LacZ could not be obtained in an active form. Large, multimeric enzymes such as LacZ are notoriously prone to aggregation and inclusion body formation during recombinant expression, even in optimized bacterial hosts. This limitation highlights a critical consideration for industrial enzyme selection: catalytic superiority in theory is insufficient if the enzyme cannot be produced in a functional and scalable form. In this context, BgaA emerges as the most promising β-galactosidase from L47, not due to superior intrinsic kinetics, but because it retains measurable activity when produced as inclusion bodies.

The observation that catalytically active enzymes can be recovered directly from washed inclusion bodies aligns with a growing body of literature redefining inclusion bodies as functional protein aggregates rather than inactive waste products [34,35,36]. Inclusion bodies have been shown to act as mechanically stable, protease-resistant reservoirs of enzymatic activity and, in some cases, as naturally immobilized biocatalysts suitable for industrial applications [37]. In this study, the use of mild washing steps with nonionic detergents allowed removal of cellular contaminants without disrupting the catalytic competence of BgaA-associated aggregates, supporting this modern view of inclusion bodies as usable biocatalytic materials.

With BgaA identified as the principal lactose-hydrolyzing enzyme, a conceptual lactose-to-tagatose bioconversion cascade can be envisioned using BgaA in combination with L-arabinose isomerase (AraA). While BgaA efficiently generates galactose from lactose, the isomerization of galactose to tagatose by AraA represents the main bottleneck of the process. Under the conditions tested, AraA yielded approximately 18% tagatose from 100 mM galactose, well below both the theoretical equilibrium and the conversion levels required for industrial feasibility. Similar limitations have been reported for other L-AIs, where high substrate concentrations (>500 mM) and optimized reaction conditions are required to approach equilibrium yields [11].

The limited performance of AraA suggests that future improvements should focus on enzyme and process optimization rather than lactose hydrolysis itself. Strategies may include protein engineering to enhance AraA stability and catalytic efficiency, immobilization approaches to reduce enzyme precipitation, or the use of alternative L-AIs with higher intrinsic activity or reduced metal dependence [6,11]. Notably, recent studies have demonstrated improved tagatose production using multi-enzyme cascades combining robust β-galactosidases with thermostable or metal-independent L-AIs [38], highlighting promising directions for future development.

Finally, the temperature and pH profiles of the L47 enzymes are noteworthy in the context of industrial flexibility. As a psychrotolerant isolate, L47 produces enzymes capable of retaining activity across a broad temperature range, from near-freezing conditions to moderately elevated temperatures. Crude extract assays revealed sustained β-galactosidase activity even at 4 °C and up to approximately 60 °C (Figure S1F), a desirable trait for processes requiring adaptability to variable operating conditions. Likewise, the slightly acidic pH optimum of AraA (~6.0) may facilitate integration with β-galactosidase-catalyzed lactose hydrolysis, enabling operation at a compromise pH that preserves acceptable activity for both enzymatic steps.

## 4. Materials and Methods

### 4.1. Selection of Strain of Interest

The 28 isolates from Antarctica, stored at −80 °C, were reactivated in Luria-Bertani (LB) liquid medium at 15 °C. Refresher cultures were performed under aerobic conditions, with orbital shaking at 120 rpm in a SKIR-601 system (Shin Saeng, Seoul, Republic of Korea) for 5 days. They were also cultured on LB agar at 15 °C for the same period. In isolates that showed growth on LB medium, the ability to utilize L-arabinose as the sole source of carbon and energy was evaluated. For this purpose, the microorganisms were cultured in minimal medium M9 [39] supplemented with L-arabinose, under aerobic conditions, in Erlenmeyer flasks incubated at 15 °C with shaking at 120 rpm. The ability to utilize L-arabinose as a sole carbon source was verified after 7 days of culture, determining cell growth by turbidimetry (OD_600nm_).

Similarly, the growth of strains that grew in LB medium was evaluated in M9 minimal mineral medium supplemented with lactose (2% *w*/*v*). Those strains selected for their ability to grow using lactose as the sole carbon source were plated on agar plates containing M9 minimal mineral medium supplemented with lactose (2% *w*/*v*) and 5-bromo-4-chloro-3-indolyl-β-D-galactopyranoside (X-Gal). The plates were incubated at 20 °C for 4 days or until blue colonies appeared.

### 4.2. Identification of Microorganisms by 16S rRNA Sequencing

For isolates that used L-arabinose as the sole carbon source, partial amplification of the 16S ribosomal RNA gene was performed using polymerase chain reaction (PCR). The universal primers 27F 5′-AGAGTTTGATC(A/C)TGGCTCAG-3′ and 1492R 5′-TACGG(C/T)TACCTTGTTACGACTT-3′ were used. Once the amplicon was obtained, the PCR product was purified using the Wizard^®^ SV Gel and PCR Clean-Up System (Promega©). The purified PCR product was sequenced using the MacroGen Inc. sequencing service with the primers 785F 5′-GGATTAGATACCCTGGTA-3′ and 907R 5′-CCGTCAATTC(A/C)TTT(A/G)AGTTT-3′ by capillary electrophoresis. The sequencing results were analyzed using the BLASTn database of the National Center for Biotechnology Information (NCBI), which allowed the identification of 13 bacterial isolates to the genus level. Once the strains were identified, their reference genomes deposited at the NCBI were searched for putative L-AI coding sequences.

### 4.3. Evaluation of D-Tagatose and L-Ribulose Production Using Cell Extracts from Isolates

In L47 and R61 isolates, whose genomes were found to contain putative genes for L-AI, the capacity of their cell extracts to catalyze the synthesis of L-ribulose and D-tagatose from L-arabinose and D-galactose, respectively, was evaluated.

Both isolates were grown in minimal medium M9 supplemented with L-arabinose to stimulate L-AI expression. The strains were cultured until reaching an OD_600nm_ of 1. Biomass was recovered by centrifugation at 4080 RCF and 4 °C for 15 min using a Hettich Universal 320R centrifuge machine (Hettich GmbH & Co. KG, Tuttlingen, Germany). The cell pellet was resuspended in lysis buffer (50 mM Tris-HCl, pH 7, 0.1 mM phenylmethylsulfonyl fluoride (PMSF)) and lysed by cavitation using a Sonic-650WT-V2 instrument (MRC Ltd., Holon, Israel). The ultrasonic cavitation process was carried out for 30 min at 85% amplitude, with 2 s pulses followed by 3 s rest periods. Cell debris was removed by centrifugation at 4080 RCF and 4 °C for 15 min. The supernatant (cell extract) was recovered and characterized for total protein content using the Bradford method [40], with bovine serum albumin (BSA) as the standard. The arabinose and galactose isomerization activity present in the cell extracts was determined by quantifying D-tagatose production. or L-ribulose from D-galactose or L-arabinose, respectively. As a first approach to verify the activity of the L-AI enzyme in the cell extracts, an initial reaction was carried out in an LXC thermoregulated water bath (PolyScience, Niles, IL, USA) for both isolates, at a temperature of 40 °C, using an initial substrate concentration of 300 mM, 5 mg/mL of the cell extract, 1 mM of each cofactor (CoCl_2_ and MnCl_2_), and 50 mM Tris-HCl buffer, pH 7. The reactions were conducted under shaking at 300 rpm in a total volume of 2 mL. Samples were taken at 0, 1, 2, 3, 5, and 7 days, and the reaction was stopped by incubating the samples at 99 °C for 2 min [18]. Once the presence of L-AI activity in the cell extract was confirmed, the effect of pH was evaluated by testing pH 5 and 6 (citrate buffer) and 7 and 8 (Tris-HCl), as well as the effect of temperature (4 to 60 °C) and the presence of cofactors (CoCl_2_ and MnCl_2_) on enzyme activity. The effect of these parameters was assessed by independently varying each variable. The substrates (D-tagatose or L-ribulose) and products (D-galactose or L-arabinose) were determined by high-performance liquid chromatography (HPLC) as described later in the carbohydrate analysis section.

### 4.4. Determination of β-Galactosidase Activity by o-NPG Hydrolysis

Enzyme activity in the extracts was quantified spectrophotometrically through the hydrolysis reaction of o-NPG. This reaction releases o-nitrophenol (o-NP) as a product, a compound that imparts a yellow color to the sample and exhibits absorbance at 420 nm. The optimum pH for the obtained enzymes was determined by quantifying activity within a pH range of 4 to 8. Cofactor requirements were verified by adding 0.1 mM MnCl_2_ and 0.1 mM CaCl_2_ during the activity quantification assay. All assays were performed in triplicate using an initial o-NPG concentration of 11 mM. For the purposes of this work, an international unit of β-D-galactosidase is defined as the amount of enzyme capable of hydrolyzing 1 µmol of o-NPG per minute at 20 °C.

### 4.5. Genomic DNA Isolation and Gene Identification

Genomic DNA from strain L47 was isolated using the Wizard^®^ Genomic DNA Purification Kit (Promega©) (Plasmidsaurus, Eugene, OR, USA). The quantification of purified L47 genomic DNA was performed at 260 nm on a TECAN Infinite M200 Pro multiplate reader using NanoQuant plate (Tecan Group Ltd., Männedorf, Switzerland) and its purity was assessed by means of the A260/280 ratio. This DNA was sent for sequencing and analysis to the commercial service Plasmidsaurus (Eugene, OR, USA). Sequencing was performed using Oxford Nanopore long-read technology. Subsequent analysis—including assembly, annotation, and identification of genes of interest—was carried out by the technical team of the same service, identifying the sequence corresponding to the *araA* gene in the genome by SnapGene program (version 8.0.3).

### 4.6. Genome Relatedness (ANI/dDDH) and In Silico Operon/Promoter Prediction

Taxonomic classification of Isolate L47 was initially determined using Average Nucleotide Identity (ANI) analysis against the GTDB database (release 226) genomes, as implemented in GTDB-tk software (version 2.4.1) [41]. Subsequently, a genus-level ANI analysis was performed. This involved downloading seven Genus relative genomes from the GTDB database, based on the genus assigned to Isolate L47, and calculating pairwise ANI values using fastANI software (version 1.34) [42]. These pairwise ANI values were then used to generate a heatmap visualization in R employing the ComplexHeatmap package functions [43]. Finally, digital DNA-DNA Hybridization (dDDH) was calculated between the Isolate L47 genome and the prokaryotic genome exhibiting the highest ANI value, utilizing the online web tool GGDC 3.0 [44].

Operons carrying the target genes were predicted with Operon-mapper (web version, 2021), which infers transcriptional units based on gene orientation and intergenic distances. The L47 genome was annotated with Prokka v1.14.6 [45] to confirm gene context and to identify putative sigma factor genes by homology/domain detection. Operon coordinates and orientations were validated by BLASTn using BLAST+ v2.12.0 [46]. Up to 500 bp upstream of each predicted operon were extracted and analyzed with BPROM (SoftBerr Inc., Mount Kisco, NY, USA) to identify −10/−35 motifs, estimate transcription start sites, and obtain LDF scores consistent with σ^70^-dependent promoters [47].

### 4.7. Microscopic and Genomic Characterization of the Isolate L47

Considering the L-AI enzymatic activity characteristics observed in cell extracts, in terms of pH, temperature, and cofactor dependence, the *Ewingella* sp. isolate was selected for further study. Therefore, a partial characterization of this isolate was undertaken, given the lack of detailed information on its morphology and genomics in the literature.

As a first step, morphological characterization was performed using Gram staining [48]. In addition, the strain was characterized by scanning electron microscopy (SEM) at the Electron Microscopy and Microanalysis Laboratory of the University of Chile. For sample preparation, 1 mL of a logarithmic-phase culture (OD_600nm_ ≈ 1) was taken and washed twice with 0.01 M sodium cacodylate buffer, pH 7, containing 0.15 M NaCl, and incubated for 15 min. The cells were then fixed by incubating them for 2 h at room temperature in a 1% *v*/*v* glutaraldehyde solution prepared in the same buffer (0.01 M sodium cacodylate, pH 7; 0.15 M NaCl). The samples were then washed again twice with the same 0.01 M cacodylate buffer, pH 7; 0.15 M NaCl for 15 min. Finally, the samples were dehydrated in an ascending series of ethanol (30, 50, 70, 80, 90 and 100%), coated with a thin gold film and observed in a variable pressure EVO MA10 scanning electron microscope (SEM) (Carl Zeiss) (Carl Zeiss Microscopy GmbH, Oberkochen, Germany) at 50 Pa and a voltage of 15 kV.

### 4.8. Primer Design, Gene Amplification by PCR, and Construction of Expression Vectors

For the design and synthesis of primers, the annotated genes within the sequenced genome of the *Ewingella americana* isolate, identified in the previous section, were searched. Once the sequence was confirmed, and to clone the gene into the pET-21b(+) or pET101/D-TOPO vector, primers with sequences specific to the gene of interest were generated, as shown in Table 2.

PCR reactions were performed on a Multigene™ OptiMax thermal cycler (Labnet Houston, TX, USA). Amplifications were conducted in a 50 µL volume containing the isolate’s genomic DNA, engineered primers, deoxyribonucleoside triphosphates (dNTPs), GoTaq^®^ Q5 polymerase, and GoTaq^®^ Q5 polymerase working buffer (Applied Biosystems, Foster City, CA, USA). The PCR reaction was carried out according to the following program: 95 °C for an initial 2 min, followed by 30 cycles of 95 °C for 30 s, 63 °C for 30 s, 72 °C for 50 s, and a final amplification at 72 °C for 5 min. The PCR product was visualized by electrophoresis on a 1% *w*/*v* agarose gel in pH 8 Tris-Acetate-EDTA buffer (TAE). SYBR^®^ Safe (Thermo Fisher Scientific, Waltham, MA, USA) was used for DNA staining and visualization on the transilluminator.

The PCR product was treated using the Wizard^®^ PCR preps purification system from Promega^©^ and digested with the restriction enzymes. Simultaneously, the pET-21b(+) vector was linearized using the same restriction enzymes. The digestion product and the linearized vector were contacted (at a 1:5 or 1:3 ratios (vector:PCR product)) overnight at 16 °C in the presence of T4 DNA ligase (New England Biolabs, Ipswich, MA, USA) and its corresponding ligation buffer (50 mM Tris-HCl pH 7; 10 mM MgCl_2_; 1 mM DTT; and 1 mM ATP), resulting in the recombinant pET-21b(+)-L47genes vector. Subsequently, chemocompetent *E. coli* DH5α or TOP10 cells were transformed with the respective vector pET-21b by heat shock. The transformed cells were grown in LB medium supplemented with ampicillin (50 mg/mL). Transformed colonies were verified with universal primers for the T7 region of the vector, and to verify the integrity and directionality of the insert, the recombinant plasmids were purified from confirmed colonies and amplified with the primers (Table 2) to verify the presence of the insert. Once obtained, the insert was sequenced by capillary electrophoresis at the sequencing service of the Pontificia Universidad Católica de Chile (FONDEQUIP EQM150077) using an Applied Biosystems ABI PRISM 3500 XL instrument to verify its directionality and integrity.

For the pET101/D-TOPO Champion™ vector, the forward primers were designed with a CACC sequence at the 5′ end, which is required for the directional cloning method, and the stop codon was removed from the reverse primer. In the case, purified PCR product of the *bglY* gene was used, after being purified, to ligate directly with the vector, in a 1:3 ratio following the manufacturer’s protocol, incubating the reaction for 30 min at room temperature.

### 4.9. Gene Cloning and Heterologous Expression

For *E. coli* expression, the genes were subcloned into expression plasmids (Table 3). Specifically, *bglY* was cloned into pET-101/D-TOPO (Invitrogen, Carlsbad, CA, USA) to add a C-terminal 6×His-tag, and *araA*, *bgaA* and *lacZ* were cloned into pET-21a(+) (Novagen, Madison, WI, USA) which also provides a C-terminal His-tag. These constructs (pET101/*bglY*, pET101/*araA*, pET21/*bgaA*, pET21/*lacZ*) were transformed into chemically competent *E. coli* (DH5α or TOP10 for plasmid propagation). Verified plasmids were then transformed into expression strains: *E. coli* BL21 (DE3) for the β-galactosidases and *E. coli* Rosetta (DE3) for *araA* (Rosetta provides tRNAs for rare codons, aiding expression of the *araA* gene which had several rare codons).

Expression of recombinant enzymes in *E. coli* was induced with isopropyl β-D-1-thiogalactopyranoside (IPTG). For *araA* in Rosetta (DE3), cultures were grown at 37 °C to OD_600nm_ ~0.6, then induced with 0.5 or 1 mM IPTG and incubated overnight at 37 °C to enhance soluble yield. For the β-galactosidases in BL21 (DE3), induction was similarly done at OD_600nm_ ~0.6 with 1 mM IPTG, followed by overnight expression at 18 °C. In all cases, uninduced control cultures were maintained in parallel. After expression, cells were harvested by centrifugation and resuspended in lysis buffer (20 mM Tris–HCl, pH 7 or 8, 300 mM NaCl, and 1 mM PMSF). Cells were lysed by sonication on ice.

For AraA, most of the protein was found in the soluble fraction after centrifugation (15,000× *g*, 30 min, 4 °C). For the β-galactosidases, a large portion of each recombinant protein was found in insoluble inclusion bodies, especially for BgaA, BglY, and LacZ expressed in *E. coli*. As a result, we prepared both soluble extracts and inclusion body fractions for purification, as described below.

### 4.10. Expression and Recovery of AraA and Inclusion Body Preparation of β-Galactosidases

All target enzymes were engineered with a His-tag to facilitate purification by nickel-affinity chromatography.

For L-arabinose isomerase (AraA), the protein was produced in soluble form in *E. coli* Rosetta (DE3). The clarified supernatant from cell lysate was loaded onto a HisTrap HP Ni–NTA column (GE Healthcare, Chicago, IL, USA) pre-equilibrated with binding buffer (20 mM Tris–HCl, 300 mM NaCl, 10 mM imidazole, pH 8.0). After washing the column with buffer containing 50 mM imidazole to remove non-specific proteins, AraA was eluted using 250 mM imidazole. Eluted fractions were pooled and dialyzed into storage buffer (50 mM HEPES, 100 mM NaCl, pH 7.5). The final preparation showed high purity on SDS-PAGE and yielded approximately 10 mg/L of culture.

In contrast, expression of the β-galactosidases (BgaA, BglY, LacZ) in soluble form was negligible, so these enzymes were processed from inclusion bodies. After harvesting 0.5–1 L cultures, cell pellets were lysed by sonication and the insoluble fractions (inclusion body pellets) were collected by centrifugation. These pellets underwent a stepwise washing protocol to remove contaminants and facilitate future solubilization: First wash: Pellets were resuspended in 100 mM Tris–HCl buffer (pH 7.0) containing 0.5% (*v*/*v*) Triton X-100, incubated at 25 °C for 20 min, and centrifuged at 9000 RCF (4 °C, 10 min). Second wash: The pellet was resuspended in the same buffer supplemented with DNase I (25 µg/mL), incubated for 20 min at 25 °C, and centrifuged under the same conditions. Final washes: Two additional washes were performed using Tris buffer alone (no detergents or enzymes) to eliminate residual Triton or nucleases. These sequential washes yielded clean inclusion body preparations that could be stored or used directly in enzyme assays. The resulting protein yields from washed inclusion bodies were estimated at ~3–5 mg/L for BgaA and BglY, and ~1–2 mg/L for LacZ.

All purification and processing steps were evaluated by SDS-PAGE on 12% polyacrylamide gels stained with Coomassie Brilliant Blue R-250. Protein concentration was determined using the Bradford assay (Bio-Rad Laboratories, Hercules, CA, USA), with bovine serum albumin as a standard. The processed proteins were then used for enzymatic assays and activity screening.

### 4.11. Enzymatic Activity Assays

L-arabinose isomerase (AraA) activity assays: AraA activity was assessed by measuring the production of D-tagatose (or L-ribulose) from galactose (or arabinose) via cysteine–sulfuric acid or cysteine–carbazole assays, and by high-performance liquid chromatography (HPLC) for endpoint yield. For kinetic parameters, we focused on the native L-arabinose to L-ribulose reaction, which is easier to monitor continuously. Initial reaction rates were measured at 30 °C in 50 mM HEPES buffer (pH 7.5) by varying L-arabinose concentration (50–600 mM) in the presence of 1 mM MnCl_2_, which was selected based on preliminary metal-dependence assays showing maximal activation compared to Co^2+^ or Mg^2+^. We used cysteine and sulfuric acid to derivatize produced L-ribulose (which produces a distinct absorbance at 548 nm) and measured absorbance over time. Michaelis–Menten kinetics (V_max_, K_m_) for AraA were obtained by nonlinear regression of the initial velocity data. Additionally, to evaluate tagatose production, purified AraA (~0.2 mg/mL) was incubated with 100 mM D-galactose in 50 mM HEPES, pH 7.5, with 1 mM Mn^2+^ at 30 °C. After 48 h, the reaction mixture was analyzed by HPLC (Rezex RCM monosaccharide column) with refractive index detection to determine the concentration of D-tagatose produced. We also tested AraA activity across different pH values (pH 5.5 to 8.5) and with alternative metal ions (Co^2+^, Mg^2+^, or no metal, in comparison to Mn^2+^) to assess its cofactor requirements and stability, as detailed in the Supplementary Materials.

β-galactosidase activity assays: The enzymatic activity of inclusion bodies obtained from transformed *E. coli* BL21 (DE3) strains was evaluated. Analysis was performed spectrophotometrically via the o-NPG hydrolysis reaction, which releases o-NP as a product. This yellow compound exhibits absorbance at 420 nm. β-galactosidase activity assays were performed using washed inclusion bodies, as these aggregates retain catalytically active protein and avoid potential loss of activity associated with chaotropic solubilization and refolding procedures. Mn^2+^ (0.1 mM) was included as a functional divalent cation cofactor, as it can substitute for Mg^2+^ in β-galactosidase catalysis, while Ca^2+^ (1 mM) was added to support potential structural stabilization, particularly for GH42 family enzymes. Preliminary assays comparing metal conditions are shown in Supplementary Figure S5. The reaction mixture contained 100 µL of the inclusion body solution, 0.1 mM MnCl_2_ cofactor, 1 mM CaCl_2_ cofactor, 45 mM o-NPG substrate, and 100 mM Tris-HCl buffer (pH 4–pH 7). The reaction was carried out for 90 min.

To determine the kinetic parameters of BgaA, it was analyzed via the o-NPG hydrolysis reaction, measuring absorbance at 420 nm as described above. The reaction mixture contained 100 µL of inclusion solution, 1 mM MnCl_2_ cofactor, 10, 15, 20, 25, 30, 35, 40, 45, 50, 55, and 60 mM substrate (o-NPG), and 100 mM Tris-HCl buffer at pH 6. The reaction was carried out for 90 min.

The experiments were conducted on the TECAN Infinite M200 Pro multi-reader using a NanoQuant plate (TECAN) (Tecan Group Ltd., Männedorf, Switzerland). The assay was performed in triplicate. For the purposes of this work, one international unit of β-Gal is defined as the amount of enzyme capable of hydrolyzing 1 µmol of o-NPG per minute at room temperature.

### 4.12. Computational Sequence and Structural Analysis

Homology modeling: Three-dimensional models of the L47 enzymes (LacZ, BgaA, BglY, AraA) were constructed to analyze their structural features. For each enzyme, we identified suitable template structures via BLAST and HHsearch against the Protein Data Bank [49]. The best templates were: *E. coli* LacZ (PDB ID: 1JYN, 50% identity [7]) for L47 LacZ; a *Bacillus* GH42 β-galactosidase (PDB ID: 3TTS, 55% identity [26]) for L47 BglY; an intracellular *Geobacillus* GH42 β-galactosidase (GanB, PDB ID: 4OIF, 48% identity [50]) for L47 BgaA; and *E. coli* L-arabinose isomerase (PDB ID: 2AJT, 70% identity [25]) for L47 AraA. Models were built using Alphafold server [51] and MODELLER 10.4 [52], generating 100 candidate structures for each enzyme. Models were evaluated by their DOPE score [53] and verified for stereochemical quality and fold accuracy using tools like VERIFY3D and PROCHECK [54]. The top-ranking model for each enzyme was selected for further analysis. Figures of protein structures were prepared with PyMOL v2.5 and VMD v1.9 [55].

Substrate docking: Lactose was docked into the β-galactosidase models (LacZ, BgaA, BglY) and D-galactose into the AraA model to examine substrate binding modes. We used AutoDock Vina [56] software to generate plausible poses of lactose in the enzyme active sites. For each β-galactosidase, the search space was defined around the catalytic cleft containing the two key glutamates. For AraA, docking of galactose considered both closed-ring and open-chain forms. Top-scoring poses were inspected for consistency with known binding in homologous structures (e.g., lactose in *E. coli* LacZ, arabinose in *E. coli* AraA). The best pose for each enzyme–substrate pair was used as the starting conformation for simulations.

Molecular dynamics (MD) simulations: To refine the docking poses and assess stability, we carried out MD simulations (100 ns each) of the enzyme–substrate complexes. Each complex was placed in a cubic water box with 150 mM NaCl. Simulations were performed with the NAMD 2.14 engine [57] using the CHARMM36 force field for proteins and carbohydrates. After energy minimization and equilibration (with gradual relaxation of position restraints on the protein), production runs of 100 ns were conducted in the NPT ensemble at 300 K. Coordinates were saved every 2 ps. We monitored the root-mean-square deviation (RMSD) of backbone atoms over time to ensure the systems reached equilibrium and computed the root-mean-square fluctuation (RMSF) for key active-site residues to evaluate flexibility.

Binding free energy calculations: To estimate relative binding affinities of lactose/galactose to the enzymes, we performed endpoint free energy calculations using the Molecular Mechanics/Generalized Born Surface Area (MM/GBSA) method [58]. From each 100 ns MD trajectory, we extracted snapshots (every 10 ns in the equilibrated phase) and calculated the ΔG of binding for the substrate to the enzyme using the GB model and surface area term for solvation. The average ΔG_bind_ and standard deviation were obtained for each enzyme–substrate complex. While absolute values from MM/GBSA have limited accuracy, the comparative rankings provide insight into which enzyme is predicted to bind the substrate most tightly. For the free energy, 3000 frames from the simulation were taken for LacZ, 1500 frames for both BgaA and BglY, and 2500 frames for AraA.

## 5. Conclusions

This study establishes the Antarctic isolate L47 as *Ewingella americana* and demonstrates that its genome encodes a rare set of catabolic enzymes relevant to lactose valorization: one L-arabinose isomerase (AraA) and three β-galactosidases (BgaA, BglY, and LacZ). Functional assays support three main conclusions. First, recombinant AraA is catalytically active on D-galactose and requires Mn^2+^, but the tagatose yield achieved under the tested conditions (~18% from 100 mM galactose at 48 h) indicates that isomerization efficiency and/or stability remain limiting for an industrially competitive process. Second, the β-galactosidases were largely insoluble in *E. coli*; when activity was evaluated from washed inclusion-body preparations, BgaA showed the most consistent β-galactosidase activity, while BglY and LacZ did not yield reproducible activity in the current workflow. Third, the observed mismatch between in silico expectations and experimental performance highlights that practical enzyme utility depends strongly on expression and recoverability in a production host. Together, these findings position BgaA as the most tractable lactose-hydrolyzing component from L47 at this stage and identify AraA as the key bottleneck for improving overall lactose-to-tagatose conversion. Future work should prioritize enhancing AraA performance (enzyme engineering and/or process intensification) and transferring BgaA production to a GRAS host to enable scalable and food-compatible bioconversion of lactose-rich streams.

## Figures and Tables

**Table 1:** Calculated ligand binding free energies (ΔG_bind_) from MM/GBSA analysis of MD simulations. The standard deviation (SD) indicating fluctuations. More negative values imply stronger binding affinity.

Enzyme	Free Energy (kcal/mol)	Standard Deviation (kcal/mol)
AraA	−12.49	0.10
LacZ	−28.28	0.15
BgaA	−24.74	0.08
BglY	−7.43	0.06

**Table 2:** Primers used in this work. RE: restriction enzyme.

Gen	Primer		Sequence 5′ → 3′	T_m_ (°C)	RE *
*araA* (pET21)	F	F_AraA	AA**CATATG**GACGCGTTTAAACA	62.0	*Nde*I
	R	R_AraA	TT**AAGCTT**TTTTGCGCAAAGTTTAAA	62.0	*Hind*III
*bgaA* (pET21)	F	F_BgaA	AAT**AAGCTT**AGCCAATTATATTATGGC	52.0	*Hind*III
	R	R_BgaA	ATA**CTCGAG**GCTTGTGACGAT	55.7	*Xho*I
*lacZ* (pET21)	F	F_LacZ	TT**AAGCTT**AATCCTGAAACCGCT	54.3	*Hind*III
	R	R_LacZ	AA**CTCGAG**ATTTTCTGCGTTT	52.4	*Xho*I
*bglY* (pET101)	F	F_Bgly.T	CACCATGAATAAATTTCCTCCTTTGAGTGC	58.2	
	R	R_BglY.T	TGCCAGTCGGCGGGA	60.5	
16S (positive control)	F	16S_8F	AGAGTTTGATCCTGGCTCAG	51.5	
	R	16S_1492R	ACGGCTACCTTGTTACGACTT	50.1	
T7 terminador	R	T7 ter	GCTAGTTATTGCTCAGCGG	53.4	

* RE: restriction enzyme site included in the primer sequence.

**Table 3:** Bacterial strains used in this work.

Strain	Main Features	Source or Reference
*E. coli* DH5α	*end*A1, *rec*A1, *gyr*A96, *thi*, *hsd*R17, *rel*A1, *sup*E44, D*lac*U169, 80 l*ac*ZDM1	Microbiology Molecular Laboratory
*E. coli* Rosetta (DE3)	F^-^ *omp*T *hsd*S_B_(r_B_-m_B_-) *gal dcm lac*Y1(DE3) pRARE(Cm^R^)	Microbiology Molecular Laboratory
*E. coli* TOP 10	F- *mcrA* Δ(*mrr-hsd*RMS-*mcr*BC) Φ80*lac*ZΔM15 Δ *lac*X74 *rec*A1 *ara*D139 Δ(*araleu*)7697 *gal*U *gal*K *rps*L (StrR) *end*A1 *nup*G	Invitrogen^®^
*E. coli* BL21 (DE3)	F—*omp*T *hsdS*_B_(r_B_-m_B_-) gal *dcm* (DE3)	Invitrogen^®^
Isolate L47	Identified as *Ewingella americana*	Microbiology Molecular Laboratory
Rosetta pET21*/araA*	*E. coli* Rosetta (DE3) transformed with the pET21/ara*A* plasmid	This work
BL21 pET21/*bgaA*	*E. coli* BL21 (DE3) transformed with the pET21/*bgaA* plasmid	This work
BL21 pET101-D/*bglY*	*E. coli* BL21 (DE3) transformed with the pET101-D/b*glY* plasmid	This work
BL21 pET21/*lacZ*	*E. coli* BL21 (DE3) transformed with the pET21/*lacZ* plasmid	This work

## Data Availability

All data generated or analyzed during this study are included in this published article and the Supplementary Materials Files.

## Supplementary Materials

The following supporting information can be downloaded at: https://www.mdpi.com/article/10.3390/ijms27021128/s1.

## References

[1] Díaz M.C., Glaves A. (2020). Relación Entre Consumo de Alimentos Procesados, Ultraprocesados y Riesgo de Cáncer: Una Revisión Sistemática. Rev. Chil. Nutr..

[2] Kroyer G.T. (1995). Impact of Food Processing on the Environment—An Overview. LWT Food Sci. Technol..

[3] Kokkiligadda A., Beniwal A., Saini P., Vij S. (2016). Utilization of Cheese Whey Using Synergistic Immobilization of β-Galactosidase and *Saccharomyces cerevisiae* Cells in Dual Matrices. Appl. Biochem. Biotechnol..

[4] Barros M.V., Salvador R., Do Prado G.F., De Francisco A.C., Piekarski C.M. (2021). Circular Economy as a Driver to Sustainable Businesses. Clean. Environ. Syst..

[5] Mishra B., Mohanta Y.K., Reddy C.N., Reddy S.D.M., Mandal S.K., Yadavalli R., Sarma H. (2023). Valorization of Agro-Industrial Biowaste to Biomaterials: An Innovative Circular Bioeconomy Approach. Circ. Econ..

[6] Kim P. (2004). Current Studies on Biological Tagatose Production Using L-Arabinose Isomerase: A Review and Future Perspective. Appl. Microbiol. Biotechnol..

[7] Juers D.H., Heightman T.D., Vasella A., McCarter J.D., Mackenzie L., Withers S.G., Matthews B.W. (2001). A Structural View of the Action of *Escherichia coli* (*Lac*Z) β-Galactosidase. Biochemistry.

[8] Bultema J.B., Kuipers B.J.H., Dijkhuizen L. (2014). Biochemical Characterization of Mutants in the Active Site Residues of the Β-galactosidase Enzyme of *Bacillus circulans* ATCC 31382. FEBS Open Bio.

[9] Brás N.F., Fernandes P.A., Ramos M.J. (2010). QM/MM Studies on the β-Galactosidase Catalytic Mechanism: Hydrolysis and Transglycosylation Reactions. J. Chem. Theory Comput..

[10] Rico-Díaz A., Ramírez-Escudero M., Vizoso-Vázquez Á., Cerdán M.E., Becerra M., Sanz-Aparicio J. (2017). Structural Features of *Aspergillus niger* Β-galactosidase Define Its Activity against Glycoside Linkages. FEBS J..

[11] Shehata H.M., Abd El-Ghany M.N., Hamdi S.A., Abomughaid M.M., Ghaleb K.I., Kamel Z., Farahat M.G. (2023). Characterization of a Metallic-Ions-Independent L-Arabinose Isomerase from Endophytic *Bacillus amyloliquefaciens* for Production of D-Tagatose as a Functional Sweetener. Fermentation.

[12] Mangiagalli M., Lotti M. (2021). Cold-Active β-Galactosidases: Insight into Cold Adaptation Mechanisms and Biotechnological Exploitation. Mar. Drugs.

[13] Vera C., Guerrero C., Aburto C., Cordova A., Illanes A. (2020). Conventional and Non-Conventional Applications of β-Galactosidases. Biochim. Biophys. Acta BBA-Proteins Proteom..

[14] Schmidt M., Stougaard P. (2010). Identification, Cloning and Expression of a Cold-active Β-galactosidase from a Novel Arctic Bacterium, *Alkalilactibacillus ikkense*. Environ. Technol..

[15] Pawlak-Szukalska A., Wanarska M., Popinigis A.T., Kur J. (2014). A Novel Cold-Active β-d-Galactosidase with Transglycosylation Activity from the Antarctic *Arthrobacter* sp. 32cB–Gene Cloning, Purification and Characterization. Process. Biochem..

[16] Núñez-Montero K., Salazar R., Santos A., Gómez-Espinoza O., Farah S., Troncoso C., Hoffmann C., Melivilu D., Scott F., Barrientos Díaz L. (2021). Antarctic Rahnella Inusitata: A Producer of Cold-Stable β-Galactosidase Enzymes. Int. J. Mol. Sci..

[17] Wanarska M., Kur J. (2012). A Method for the Production of D-Tagatose Using a Recombinant *Pichia pastoris* Strain Secreting β-D-Galactosidase from *Arthrobacter chlorophenolicus* and a Recombinant L-Arabinose Isomerase from *Arthrobacter* sp. 22c. Microb. Cell Factories.

[18] Rhimi M., Bajic G., Ilhammami R., Boudebbouze S., Maguin E., Haser R., Aghajari N. (2011). The Acid-Tolerant L-Arabinose Isomerase from the Mesophilic Shewanella Sp. ANA-3 Is Highly Active at Low Temperatures. Microb. Cell Factories.

[19] Xu W., Fan C., Zhang T., Jiang B., Mu W. (2016). Cloning, Expression, and Characterization of a Novel l-Arabinose Isomerase from the Psychrotolerant Bacterium *Pseudoalteromonas haloplanktis*. Mol. Biotechnol..

[20] Weimberg R., Doudoroff M. (1955). The Oxidation of L-Arabinose by *Pseudomonas saccharophila*. J. Biol. Chem..

[21] Dagley S., Trudgill P. (1965). The Metabolism of Galactarate, D-Glucarate and Various Pentoses by Species of *Pseudomonas*. Biochem. J..

[22] Dahms A.S., Anderson R.L. (1969). 2-Keto-3-Deoxy-L-Arabonate Aldolase and Its Role in A New Pathway of L-Arabinose Degradation. Biochem. Biophys. Res. Commun..

[23] Malfanova N., Kamilova F., Validov S., Chebotar V., Lugtenberg B. (2013). Is L-Arabinose Important for the Endophytic Lifestyle of *Pseudomonas* spp.?. Arch. Microbiol..

[24] Spröer C., Mendrock U., Swiderski J., Lang E., Stackebrandt E. (1999). The Phylogenetic Position of *Serratia*, *Buttiauxella* and Some Other Genera of the Family Enterobacteriaceae. Int. J. Syst. Evol. Microbiol..

[25] Manjasetty B.A., Chance M.R. (2006). Crystal Structure of *Escherichia coli* L-Arabinose Isomerase (ECAI), The Putative Target of Biological Tagatose Production. J. Mol. Biol..

[26] Maksimainen M., Paavilainen S., Hakulinen N., Rouvinen J. (2012). Structural Analysis, Enzymatic Characterization, and Catalytic Mechanisms of Β-galactosidase from *Bacillus circulans* sp. *Alkalophilus*. FEBS J..

[27] Zhou Z., He N., Han Q., Liu S., Xue R., Hao J., Li S. (2021). Characterization and Application of a New β-Galactosidase Gal42 From Marine Bacterium *Bacillus* sp. BY02. Front. Microbiol..

[28] Jacob F., Monod J. (1961). Genetic Regulatory Mechanisms in the Synthesis of Proteins. J. Mol. Biol..

[29] Schleif R. (2010). AraC Protein, Regulation of the l-Arabinose Operon in *Escherichia coli*, and the Light Switch Mechanism of AraC Action. FEMS Microbiol. Rev..

[30] Van Camp B.M., Crow R.R., Peng Y., Varela M.F. (2007). Amino Acids That Confer Transport of Raffinose and Maltose Sugars in the Raffinose Permease (RafB) of *Escherichia coli* as Implicated by Spontaneous Mutations at Val-35, Ser-138, Ser-139, Gly-389 and Ile-391. J. Membr. Biol..

[31] Strey J., Wittchen K.D., Meinhardt F. (1999). Regulation of β-Galactosidase Expression in *Bacillus megaterium* DSM319 by a XylS/AraC-Type Transcriptional Activator. J. Bacteriol..

[32] Watzlawick H., Morabbi Heravi K., Altenbuchner J. (2016). Role of the *ganSPQAB* Operon in Degradation of Galactan by *Bacillus subtilis*. J. Bacteriol..

[33] Ventura S., Villaverde A. (2006). Protein Quality in Bacterial Inclusion Bodies. Trends Biotechnol..

[34] Ramón A., Señorale-Pose M., Marín M. (2014). Inclusion Bodies: Not That Bad…. Front. Microbiol..

[35] Flores S.S., Nolan V., Perillo M.A., Sánchez J.M. (2019). Superactive β-Galactosidase Inclusion Bodies. Colloids Surf. B Biointerfaces.

[36] García-Fruitós E., González-Montalbán N., Morell M., Vera A., Ferraz R.M., Arís A., Ventura S., Villaverde A. (2005). Aggregation as Bacterial Inclusion Bodies Does Not Imply Inactivation of Enzymes and Fluorescent Proteins. Microb. Cell Factories.

[37] Krauss U., Jäger V.D., Diener M., Pohl M., Jaeger K.-E. (2017). Catalytically-Active Inclusion Bodies—Carrier-Free Protein Immobilizates for Application in Biotechnology and Biomedicine. J. Biotechnol..

[38] Aburto C., Vera C., Arenas F., Illanes A., Guerrero C. (2024). One-Pot Production of Tagatose Using l-Arabinose Isomerase from *Thermotoga maritima* and β-Galactosidase from *Aspergillus oryzae*. LWT.

[39] Miller J.H. (1977). Experiments in Molecular Genetics.

[40] Bradford M.M. (1976). A Rapid and Sensitive Method for the Quantitation of Microgram Quantities of Protein Utilizing the Principle of Protein-Dye Binding. Anal. Biochem..

[41] Chaumeil P.-A., Mussig A.J., Hugenholtz P., Parks D.H. (2020). GTDB-Tk: A Toolkit to Classify Genomes with the Genome Taxonomy Database. Bioinformatics.

[42] Jain C., Rodriguez-R L.M., Phillippy A.M., Konstantinidis K.T., Aluru S. (2018). High Throughput ANI Analysis of 90K Prokaryotic Genomes Reveals Clear Species Boundaries. Nat. Commun..

[43] Gu Z. (2022). Complex Heatmap Visualization. iMeta.

[44] Meier-Kolthoff J.P., Carbasse J.S., Peinado-Olarte R.L., Göker M. (2022). TYGS and LPSN: A Database Tandem for Fast and Reliable Genome-Based Classification and Nomenclature of Prokaryotes. Nucleic Acids Res..

[45] Seemann T. (2014). Prokka: Rapid Prokaryotic Genome Annotation. Bioinformatics.

[46] Camacho C., Coulouris G., Avagyan V., Ma N., Papadopoulos J., Bealer K., Madden T.L. (2009). BLAST+: Architecture and Applications. BMC Bioinform..

[47] Krusenstjerna A.C., Saylor T.C., Arnold W.K., Tucker J.S., Stevenson B. (2023). *Borrelia burgdorferi* DnaA and the Nucleoid-Associated Protein EbfC Coordinate Expression of the *dnaX-ebfC* Operon. J. Bacteriol..

[48] Gram H.C. (1884). Ueber die isolirte Färbung der Schizomyceten in Schnitt- und Trockenpräparaten. Fortschritte Med..

[49] Berman H.M. (2000). The Protein Data Bank. Nucleic Acids Res..

[50] Solomon H.V., Tabachnikov O., Feinberg H., Govada L., Chayen N.E., Shoham Y., Shoham G. (2013). Crystallization and Preliminary Crystallographic Analysis of GanB, a GH42 Intracellular β-Galactosidase from *Geobacillus stearothermophilus*. Acta Crystallograph. Sect. F Struct. Biol. Cryst. Commun..

[51] Jumper J., Evans R., Pritzel A., Green T., Figurnov M., Ronneberger O., Tunyasuvunakool K., Bates R., Žídek A., Potapenko A. (2021). Highly Accurate Protein Structure Prediction with AlphaFold. Nature.

[52] Webb B., Sali A., Kihara D. (2014). Protein Structure Modeling with MODELLER. Protein Structure Prediction.

[53] Shen M., Sali A. (2006). Statistical Potential for Assessment and Prediction of Protein Structures. Protein Sci..

[54] Dym O., Eisenberg D., Yeates T.O., Rossmann M.G., Arnold E. (2006). Detection of Errors in Protein Models. International Tables for Crystallography.

[55] Humphrey W., Dalke A., Schulten K. (1996). VMD: Visual Molecular Dynamics. J. Mol. Graph..

[56] Trott O., Olson A.J. (2010). AutoDock Vina: Improving the Speed and Accuracy of Docking with a New Scoring Function, Efficient Optimization, and Multithreading. J. Comput. Chem..

[57] Phillips J.C., Hardy D.J., Maia J.D.C., Stone J.E., Ribeiro J.V., Bernardi R.C., Buch R., Fiorin G., Hénin J., Jiang W. (2020). Scalable Molecular Dynamics on CPU and GPU Architectures with NAMD. J. Chem. Phys..

[58] Godschalk F., Genheden S., Söderhjelm P., Ryde U. (2013). Comparison of MM/GBSA Calculations Based on Explicit and Implicit Solvent Simulations. Phys. Chem. Chem. Phys..

