# Supplementary material for: Discovery and Preliminary Characterization of Lactose-Transforming Enzymes in Ewingella americana L47: A Genomic, Biochemical, and In Silico Approach
Source: Int J Mol Sci. 2026 Jan 22;27(2):1128. doi: 10.3390/ijms27021128 (PMC12842569; doi:10.3390/ijms27021128)
Supplement: Supplementary file 1 [file ijms-27-01128-s001.zip › ijms-4065890-supplementary.pdf]

Supplementary Figures and Table

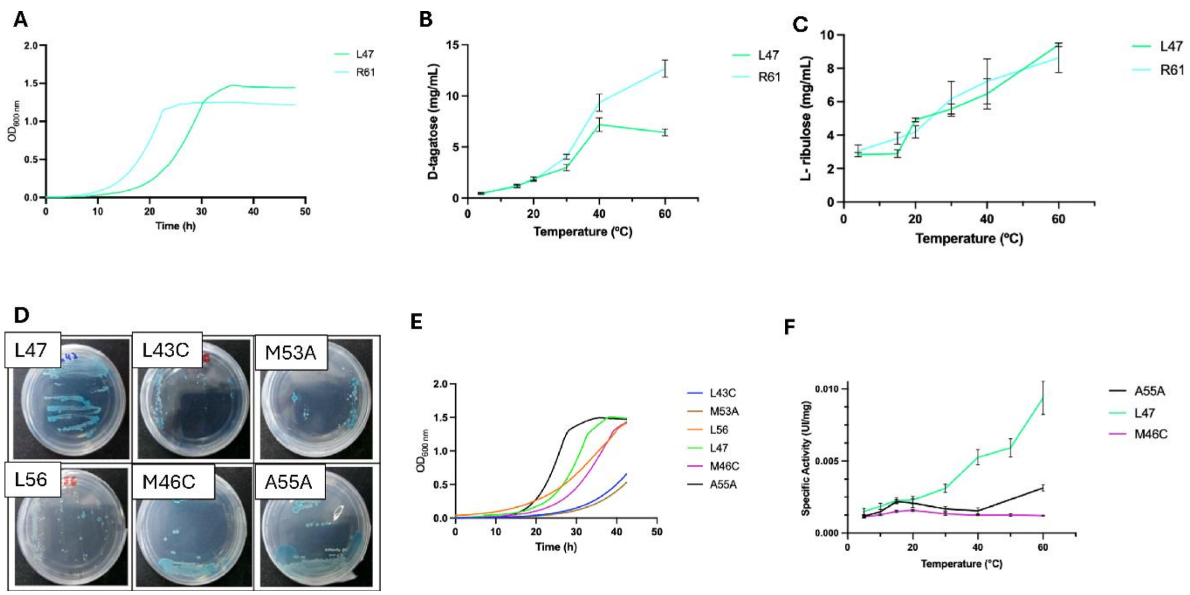

Figure S1. Analysis of the growth of environmental strains in media supplemented with different substrates, and determination of L-AI and β-gal enzyme activities in extracts. (**A**) Growth kinetics of selected strains in minimal medium M9 supplemented with L-arabinose at 15 °C. (**B**) Effect of temperature on the specific activity for the D-tagatose synthesis reaction in crude extracts. (**C**) Effect of temperature on the specific activity for the L-ribulose synthesis reaction in crude extracts. (**D**) Selected strains grown in solid minimal medium M9 supplemented with lactose (2% w/v) and X-Gal. (**E**) Growth kinetics of selected strains in minimal medium M9 supplemented with lactose (2% w/v) at 20 °C. (**F**) Effect of temperature on the specific activity for the o-NPG hydrolysis reaction in crude extracts.

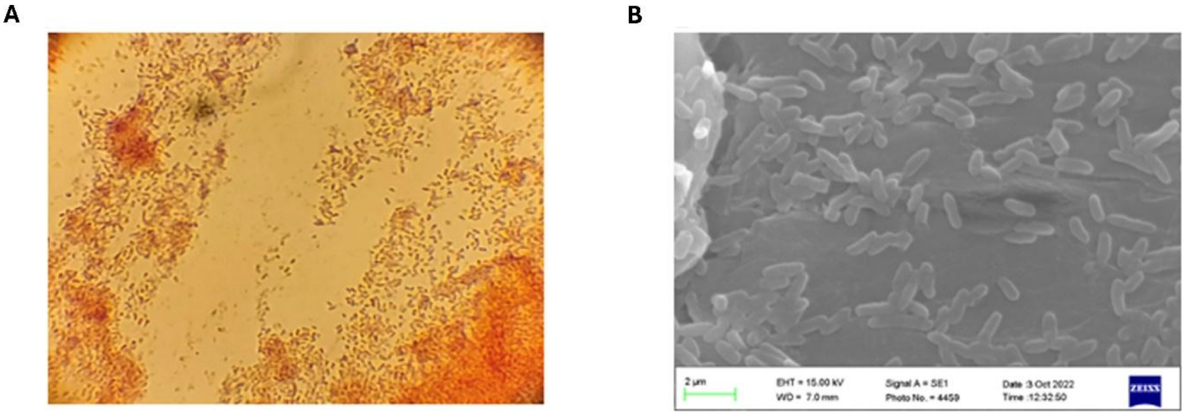

Figure S2. Microscopic characterization of strain L47. (**A**) Bright-field optical microscopy with Gram staining 100X. (**B**) Scanning electron microscopy 50000X.

Table S1. Biochemical characterization of isolate L47.

Test	Result
Urea (URE)	Negative
Arginine (ADH)	Negative
Ornithine (ODC)	Negative
Lysine (LDC)	Negative
Aliphatic thiol (TET)	Negative
Fatty acid esters (LIP)	Negative
Sugar aldehydes (KSF)	Positive
Sorbitol (SLB)	Positive
p-Nitrophenyl-β, D-glucuronide (GUR)	Negative
o-Nitrophenyl-β, D- galactoside (o-NPG)	Positive
p-Nitrophenyl-β, D-glucoside (βGLU)	Positive
p-Nitrophenyl-N-acetyl-β, D- xyloside (βXYL)	Negative
p-Nitrophenyl-N-acetyl-β, D- glucosamine (NAG)	Positive
Malonato (MAL)	Negative
Proline-β-naphthylamide (PRO)	Positive
γ -Glutamyl-β-naphthylamide (GGT)	Positive
Pyrrolidonyl -β-naphthylamide (PYR)	Positive
Adonitol (ADON)	Negative
Tryptophane (IND)	Negative

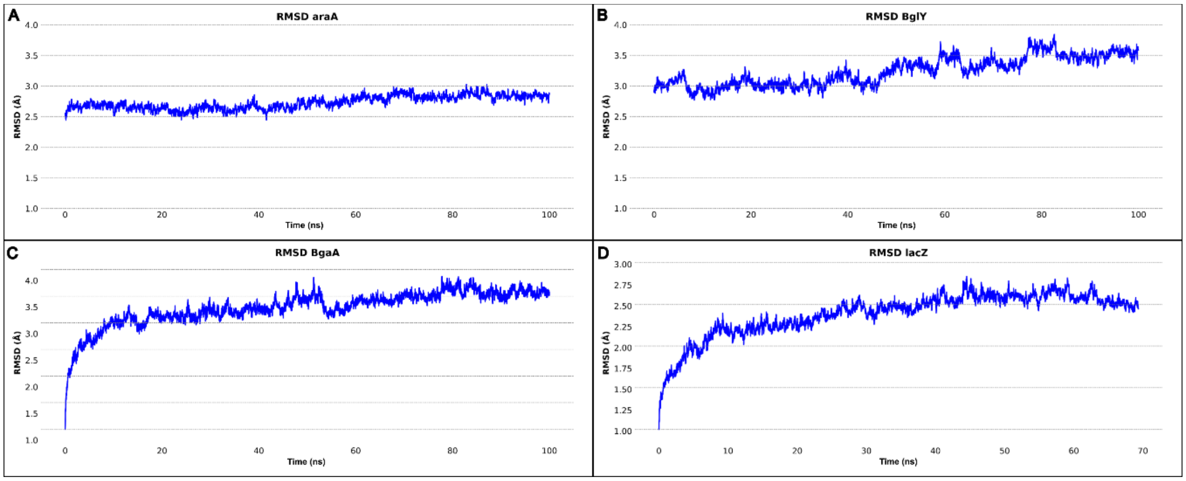

Figure S3. RMSD results of the different enzymes. On the Y-axis, the distance in Å can be seen, and on the X-axis the time in nanoseconds. In (**A**), AraA is shown; in (**B**), BgaA; in (**C**), BglY; and (**D**), LacZ.

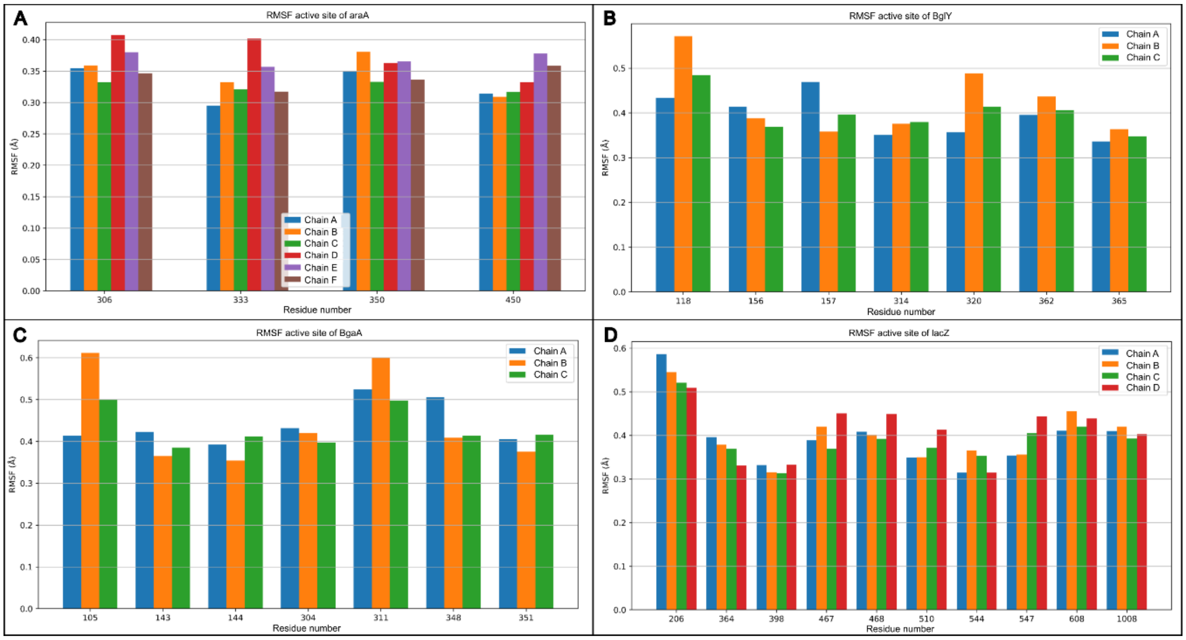

Figure S4. RMSF (Root Mean Square Fluctuation) of active-site residues in the MD simulations of each enzyme. (**A**) RMSF (Å) of key catalytic and substrate-binding residues in AraA (monomer simulation but labeled as chain A for reference) – showing minimal fluctuation of Glu332, Glu346, His104, His173 (all RMSF <0.3 Å). (**B**) RMSF of active-site residues in BgaA – note higher RMSF (~1 Å) for the loop containing Trp351 (orange bar) compared to other catalytic residues (Glu160, Glu345 ~0.5 Å). (**C**) BglY active-site residues – mostly low RMSF (<0.5 Å) except a slight bump for a loop adjacent to Thr320. (**D**) LacZ active-site residues – very low RMSF across the board (<0.4 Å for Glu461, Glu645, etc.), indicating a rigid binding pocket.

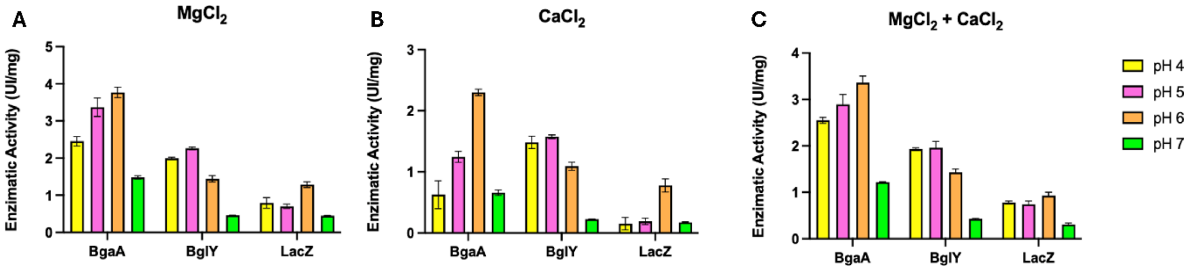

Figure S5. β-galactosidase activities (o-NPG hydrolysis) evaluated in the inclusion bodies of transformant strains with recombinant *E. coli* BL21 vectors. The assay was performed at pH 4, 5, 6 and 7 in presence of different cofactors, (**A**) $MgCl_2$, (**B**) $CaCl_2$ and (**C**) $MgCl_2$ and $CaCl_2$. For the purposes of this work, an international unit of β-gal is defined as the amount of enzyme capable of hydrolyzing 1 μmol of o-NPG per minute at room temperature. Error bars represent ±SD (n=3).
